# NSUN2-mediated m^5^C modification of HBV RNA positively regulates HBV replication

**DOI:** 10.1371/journal.ppat.1011808

**Published:** 2023-12-04

**Authors:** Jiangpeng Feng, Tianmo Xu, Miao He, Jiali Li, Peipei Yao, Chengbao Ma, Shimin Yang, Zaichao Xu, Kun Yan, Xianying Chen, Hongyun Wang, Jiejie Liu, Cong Zeng, Yuchen Xia, Huan Yan, Li Zhou, Yu Chen

**Affiliations:** 1 State Key Laboratory of Virology, RNA Institute, College of Life Sciences and Frontier Science Center for Immunology and Metabolism, Wuhan University, Wuhan, China; 2 Animal Bio-safety Level III Laboratory/Institute for Vaccine Research, Wuhan University School of Medicine, Wuhan, China; 3 School of Medicine, Sun Yat-sen University, Guangzhou, China; 4 Wuhan University Taikang Medical School (School of Basic Medical Sciences), Wuhan, China; 5 School of Basic Medical Sciences, Fudan University, Shanghai, China; University of Wisconsin-Madison, UNITED STATES

## Abstract

Chronic hepatitis B virus (HBV) infection is a major cause of liver cirrhosis and liver cancer, despite strong prevention and treatment efforts. The study of the epigenetic modification of HBV has become a research hotspot, including the *N*6-methyladenosine (m^6^A) modification of HBV RNA, which plays complex roles in the HBV life cycle. In addition to m^6^A modification, 5-methylcytosine (m^5^C) is another major modification of eukaryotic mRNA. In this study, we explored the roles of m^5^C methyltransferase and demethyltransferase in the HBV life cycle. The results showed that m^5^C methyltransferase NSUN2 deficiency could negatively regulate the expression of HBV while m^5^C demethyltransferase TET2 deficiency positively regulates the expression of HBV. Subsequently, we combined both *in vitro* bisulfite sequencing and high-throughput bisulfite sequencing methods to determine the distribution and stoichiometry of m^5^C modification in HBV RNA. Two sites: C2017 and C131 with the highest-ranking methylation rates were identified, and mutations at these two sites could lead to the decreased expression and replication of HBV, while the mutation of the “fake” m^5^C site had no effect. Mechanistically, NSUN2-mediated m^5^C modification promotes the stability of HBV RNA. In addition, compared with wild-type HepG2-NTCP cells and primary human hepatocytes, the replication level of HBV after NSUN2 knockdown decreased, and the ability of the mutant virus to infect and replicate in wild-type HepG2-NTCP cells and PHHs was substantially impaired. Similar results were found in the experiments using C57BL/6JGpt-*Nsun2*^+/-^ mice. Interestingly, we also found that HBV expression and core protein promoted the endogenous expression of NSUN2, which implied a positive feedback loop. In summary, our study provides an accurate and high-resolution m^5^C profile of HBV RNA and reveals that NSUN2-mediated m^5^C modification of HBV RNA positively regulates HBV replication by maintaining RNA stability.

## Author summary

The study of the epigenetic modification of viral RNA has become a research hotspot, including the *N*6-methyladenosine (m^6^A) modification and 5-methylcytosine (m^5^C). However, compared with m^6^A modification, fewer studies on RNA m^5^C modification regulating viruses have been reported. This is partly due to the low abundance of m^5^C modification in mRNA, and the limits of sequencing technology, which hinder the identification of accurate and repeatable m^5^C landscape of virus RNA. Here, we discovered that depletion of NSUN2, an m^5^C methyltransferase, could negatively regulate the replication of hepatitis B virus (HBV). Importantly, we combined *in vitro* bisulfite sequencing and high-throughput bisulfite sequencing methods to determine the distribution of HBV RNA m^5^C modification. Two sites modified by NSUN2 with the highest-ranking methylation rates were identified, and mutations of these two sites could lead to decreased replication of HBV. Mechanistically, m^5^C modification promotes the stability of HBV RNA. Taken together, we provide a high-resolution and accurate atlas of m^5^C in HBV RNA and reveal a functional role of m^5^C in viral replication, which provides potential targets for the development of antiviral drugs.

## Introduction

Chronic hepatitis infection due to hepatitis B virus (HBV) is still the main cause of hepatocellular carcinoma (HCC), and curing chronic hepatitis B (CHB) presents many challenges. Exploring the mechanisms behind its infection and replication has been an important research direction. HBV belongs to the Hepadnaviridae family. Its genome is a double-stranded relaxed circular DNA (rcDNA) of only 3.2kb. The coding frames and the transcriptional regulatory elements are highly overlapped. HBV genome DNA can transcribe five types of RNA: 3.6 kb preC RNA, 3.5 kb pgRNA, 2.4 kb preS1 RNA, 2.1 kb preS2/S RNA, and 0.7 kb X RNA. pgRNA/preC RNAs are longer than the genome length, because these two types of RNA contain terminal redundancy of sequences at their 5’ and 3’ ends, including the epsilon structures. All the HBV RNAs begin at different transcription start sites, but they end at the same transcription termination signal, which means they share a common region at the 3’ end [1].

The number of publications has recently been growing on the presence and function of RNA modifications, including *N*6-methyladenosine (m^6^A), 5-methylcytosine (m^5^C), and 2’-O- methylation, from a variety of RNA and DNA viruses [2–11]. Of these modifications, m^6^A has been studied and well-characterized, showing roles in splicing, translation, trafficking, stability, and innate immune response for viral RNA [12–16], which suggests that the post-transcriptional regulation of viruses has research import. Siddiqui et al. recently conducted in-depth studies on the regulation of HBV by m^6^A modification [17]. They identified m^6^A modification at the A1907 site of the HBV transcript, located in the epsilon structure of HBV RNA. This methylated site can play different roles at the 5’ and 3’ ends. At the 5’ end, the presence of m^6^A methylation promotes the binding of P protein to the epsilon structure and promotes pgRNA reverse-transcription. However, at the 3’ end, this site is recognized by YTHDF2 and 3, which accelerate all types of RNA degradation [17, 18]. In addition, this site modification also regulates the immune recognition and nuclear export of HBV RNA [19, 20]. Studies of m^6^A modification on HBV RNA have revealed the complex mechanism of RNA modification regulating viral expression and replication, expanded our understanding of virus-host interaction, and provided important clues and ideas for the study of other modifications.

In addition to m^6^A modification, m^5^C is another major RNA modification, and is widely distributed in a variety of eukaryotic RNAs, including rRNA, tRNA, mitochondrial RNA, and mRNA [21]. However, due to the low abundance of m^5^C modification in mRNA and the limits of sequencing technology that the treatment of bisulfite in the sequencing process leads to rigorous RNA degradation, the results of different studies on m^5^C site identification on mRNA are controversial [22]. In recent years, with the development of high-throughput bisulfite sequencing technology, studies have increasingly and accurately demonstrated the distribution of m^5^C modification in eukaryotic mRNA from different species and displayed its important roles in germ cell and embryonic development, stress response, and tumorigenesis [23–27]. The development of different types of cancer is regulated by RNA m^5^C modification, such as gastric cancer, bladder cancer, gynecologic cancers and hepatocellular carcinoma [26, 28–30]. Behind these biological events, diverse aspects of the mRNA life cycle, including pre-mRNA splicing, mRNA export, translation, and mRNA stability, are precisely regulated [26, 31–33]. To date, NSUN2 is considered the major mRNA methyltransferase of m^5^C, but NSUN6 has also been identified as a mRNA methyltransferase [25, 34, 35]. Furthermore, m^5^C modification is found on viral mRNAs, such as HIV-1 and murine leukemia virus (MLV), and more specifically on Epstein-Barr Virus (EBV), where m^5^C modifications are found on noncoding RNA. The level of m^5^C modification in HIV-1 is 10 times higher than that in cellular mRNA, which regulates translation and splicing [9]. Additionally, m^5^C modification in MLV could promote the expression and replication of viral proteins [8]. ALYREF, a m^5^C recognition protein, could recognize methylated mRNA of MLV and promote nuclear transport [36]. In addition, the m^5^C modification of EBV is located on its noncoding RNA. After knocking down NSUN2, the level of m^5^C modification decreases, but the level of EBV RNA increases, which indicates that virus replication is negatively regulated by affecting the stability of RNA [37]. The functions of m^5^C and m^6^A modifications are similar in that they can both affect viral replication and expression by mediating different RNA fate decisions. However, few studies on m^5^C modification regulating other viruses have been published, apart from those mentioned above [38]. This is partly due to the relatively low level of m^5^C modification, to the lack of conserved methylation motifs, and to the limits of sequencing technology.

In this study, we explored the role of m^5^C methyltransferase on the HBV life cycle. By knocking down or knocking out the m^5^C methyltransferase NSUN2 and demethyltransferase TET2, we found that NSUN2 positively regulates HBV expression and replication by maintaining HBV RNA stability, while TET2 has the opposite effect. Furthermore, we identified the m^5^C modification distribution of HBV RNA at single-nucleotide resolution using both *in vitro* and high-throughput bisulfite sequencing methods. And we confirmed that these m^5^C sites were modified by NSUN2. Importantly, mutation of these methylated sites substantially reduced the stability of HBV RNA and resulted in decreased expression and replication of HBV. These results were confirmed in the HepG2-NTCP and primary human hepatocytes (PHHs) infection system, also in C57BL/6JGpt-*Nsun2*^+/-^ mice. Interestingly, we also found that the endogenous expression of NSUN2 increased after HBV transfection or infection, during which HBV core protein may function as the key regulator. Taken together, this study provides a high-resolution atlas of m^5^C in HBV RNA and reveals a functional role of m^5^C in viral replication, which provides potential targets for the development of antiviral drugs.

## Results

### NSUN2 positively regulates HBV expression and replication

**Fig 1:**
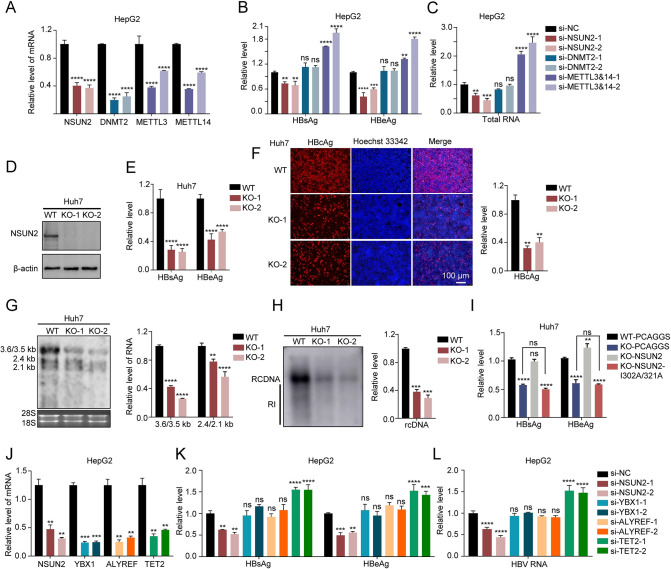
NSUN2 positively regulates HBV expression and replication by maintaining RNA stability. (A-C) siRNAs were transfected into HepG2 followed by pHBV1.3 and pSV-β-gal transfection with an interval of 12 hours. Supernatant was collected for ELISA at 48 hours post-transfection. Cells were harvested for RNA extraction or lysis. Activity of β-galactosidase in cell lysates was measured to normalize efficiency of transfection for ELISA. (*A*) qPCR results of NSUN2, DNMT2, and METTL3/14 RNA levels relative to GAPDH after siRNA transfection. (*B*) ELISA results of HBV antigen levels after NSUN2, DNMT2, and METTL3&14 depletion. (*C*) qPCR results of HBV total RNA level relative to that of GAPDH after NSUN2, DNMT2, and METTL3&14 depletion. (D-H) pHBV1.3 and pSV-β-gal were transfected into Huh7 and Huh7-NSUN2-KO cells; 48 hours later, supernatant was collected for ELISA. Cells were harvested for lysis, RNA extraction, rcDNA extraction, or immunofluorescent staining. The activity of β-galactosidase in cell lysates was measured to normalize efficiency of transfection for ELISA. (*D*) Western blot of NSUN2 protein for Huh7 and Huh7-NSUN2-KO cells. (*E*) ELISA results of HBV antigen levels after NSUN2 knockout in Huh7. (*F*) Immunofluorescent staining (left) and fluorescence intensity analysis (right) of HBcAg after NSUN2 knockout in Huh7. Relative levels were quantified using ImageJ. (*G*) Northern blot (left) and gray degree analysis (right) of HBV RNA after NSUN2 knockout in Huh7. The relative levels of HBV RNA were quantified using Quantity One. Ribosomal RNAs (28S and 18S) were used as loading controls. (*H*) Southern blot (left) and gray degree analysis (right) of HBV core-associated rcDNA in cells after NSUN2 knockout in Huh7. RI, replication intermediates. (*I*) ELISA results of HBV antigen levels with normalization in Huh7 or Huh7-NSUN2-KO cells after NSUN2 rescuing. I302A and 321A were the mutations of catalytic sites of NSUN2. PCAGGS, NSUN2, or NSUN2-I302A/321A and pHBV1.3 were transfected to Huh7 or Huh7-NSUN2-KO cells. At 48 hours later, supernatant was collected for ELISA, and cells were harvested for the detection of β-galactosidase activity. (J-L) The expression of NSUN2, YBX1, ALYREF, and TET2 in HepG2 was depleted with siRNA-mediated knockdown, followed by transfection with pHBV1.3 and pSV-β-gal. At 48 hours later, cells were harvested for RNA extraction and subsequent qPCR analysis (*J* and *L*). Supernatant was collected for ELISA (*K*). β-galactosidase activity in cell lysates was measured to normalize efficiency of transfection for ELISA. Immunoblots shown are representative of three independent experiments. Graphs show the mean ± SD derived from three independent experiments and were analyzed by one-way ANOVA (one target) or two-way ANOVA analysis (two or more targets) followed by multiple comparisons test. ns, not significant for P > 0.05, *P < 0.05, **P < 0.01, ***P< 0.001, ****P< 0.0001.

NSUN2 and DNMT2 are reported to modify viral RNA with m^5^C methylation [33]. To explore whether these two methyltransferases can affect the expression of HBV, we analyzed the changes of HBV antigens and RNA levels in NSUN2- or DNMT2-depleted HepG2 transfected with HBV expression vector (plasmid HBV1.3); we used the depletion of m^6^A methyltransferases METTL3 and METTL14 as the positive control (Fig 1*A*). The results show that the levels of HBeAg and HBsAg significantly decreased after NSUN2 depletion, whereas the antigen levels increased after METLL3 and 14 depletions (Fig 1*B*). Additionally, the level of HBV total RNA decreased after NSUN2 depletion and increased after METTL3 and 14 depletions, whereas the depletion of DNMT2 had no obvious effect (Fig 1*C*). Furthermore, we analyzed the expression and replication level of HBV in NSUN2-knockout (KO) Huh7 and HepG2 (Figs 1*D* and S1*A*). The results show that HBsAg, HBeAg, and HBcAg levels significantly decreased after NSUN2 depletion (Figs 1*E*, 1*F*, and S1*B*), and HBV RNA also remarkably decreased (Figs 1*G* and S1*C*). Further detection showed that after NSUN2 knockout, the core-associated rcDNA significantly decreased (Figs 1*H* and S1*D*), indicating that NSUN2 knockout had a negative effect on the expression and replication of HBV. Moreover, in NSUN2-rescued Huh7-NSUN2-KO, HBV antigens increased to the level in Huh7 (Figs 1*I* and S1*E*), whereas rescues with methyltransferase inactivated mutant NSUN2-I302A/321A [39], had no effect, which led us to the conclusion that NSUN2 positively regulates HBV expression by using its methylation activity.

To date, two m^5^C reader proteins, ALYREF and Y box binding protein 1 (YBX1), and one m^5^C eraser protein TET2 for mRNA, have been characterized [26, 31, 40]. In order to elucidate their potential roles in regulating HBV replication, we utilized siRNA to knock down their expression and subsequently assessed viral expression levels (Fig 1*J*, 1*K*, and 1*L*). Surprisingly, upon knockdown of these two reader proteins, no significant changes were detected in the levels of HBV antigens or RNA (Fig 1*K* and 1*L*). However, when TET2 was depleted, both the HBV antigens and RNA levels significantly increased (Fig 1*K* and 1*L*). These results suggest that m^5^C demethyltransferase TET2 negatively regulates HBV expression and imply the involvement of unidentified m^5^C reader proteins that may contribute to the regulation of HBV replication.

### NSUN2 methylated HBV RNA both *in vitro* and in the cell

**Fig 2:**
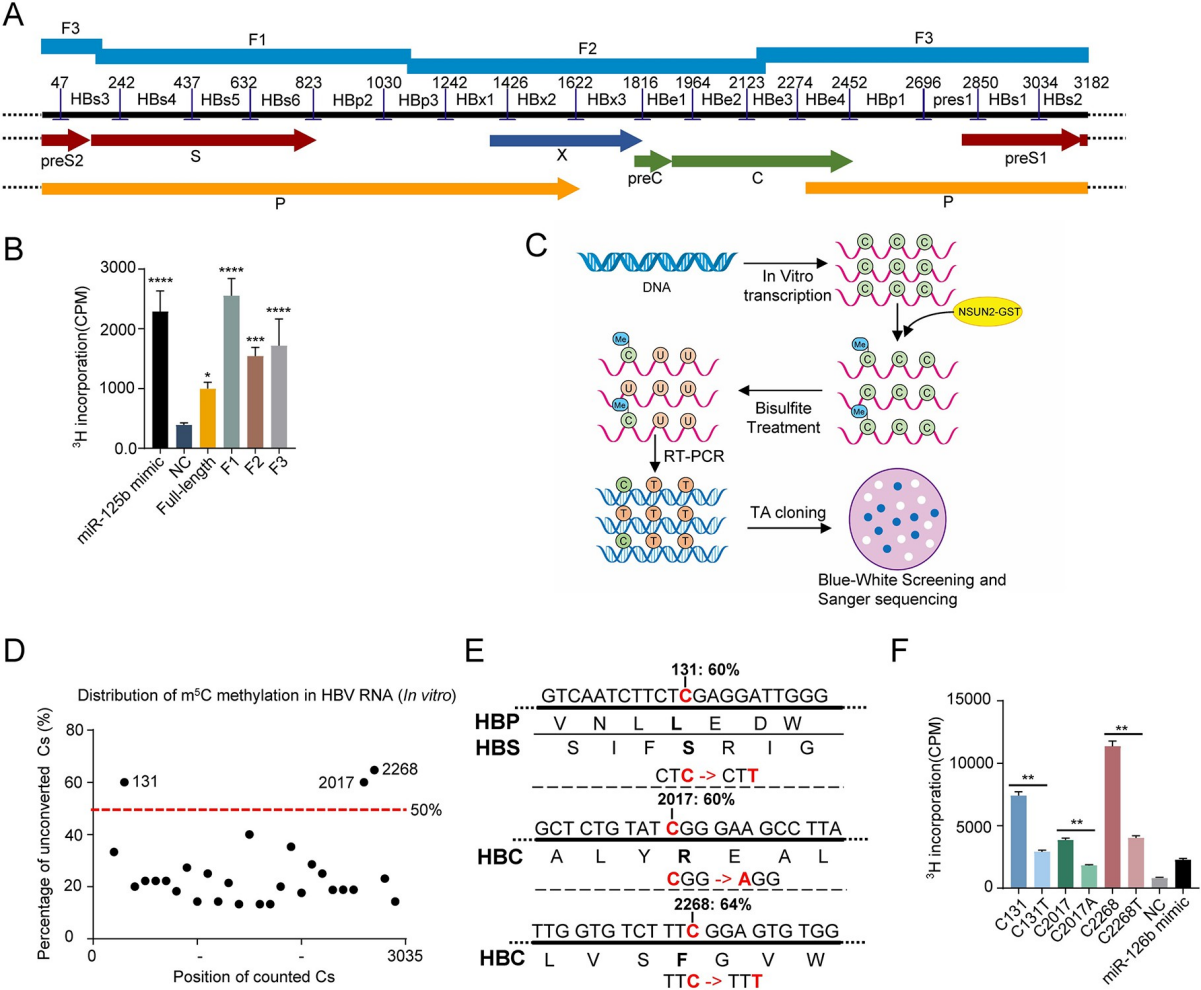
HBV RNA could be methylated by NSUN2 *in vitro*. (*A*) Schematic diagram of HBV genome with 17 fragments for *in vitro* bisulfite sequencing and 3 fragments for *in vitro* methylation. (*B*) *In vitro* m^5^C methylation assays of HBV RNA (three parts: F1, F2 and F3) using recombinant NSUN2-GST. NC: negative control. CPM: counts per minute. miR-125b mimic was used as a positive control. (*C*) Schematic diagram of *in vitro* bisulfite sequencing. All 17 parts were transcribed to RNA, then methylated with NSUN2-GST *in vitro*. Methylated RNA was subjected to bisulfite treatment and reverse-transcription PCR. Positive clones were selected through blue-white spot screening after TA cloning and then sequenced with Sanger sequencing. (*D*) Percentage of unconverted Cs in HBV RNA after treatment. The totally converted Cs were not counted. Sites with methylation level above 50% (red line) were considered potential methylated sites. (*E*) Nucleotide and amino acid sequences from three regions containing sites C131, C2017, and C2268. These three sites were mutated as shown by the red bases. Bold letters indicate the amino acid for which these bases code. (*F*) *In vitro* m^5^C methylation assays of HBV RNA after site mutation. Fragments containing these three sites were separately mutated. NC: negative control, PC: positive control, miR-125b mimic. CPM: counts per minute. Graphs show the mean ± SD derived from three independent experiments and were analyzed by unpaired Student’s t test or one-way ANOVA followed by multiple comparisons test. ns, not significant for P > 0.05, *P < 0.05, **P < 0.01, ***P< 0.001, ****P< 0.0001.

We proved that NSUN2 could regulate HBV expression and replication. To determine whether NSUN2 functioned by methylating HBV RNA, we transcribed the full length and three parts of HBV pgRNA *in vitro*, separately (Fig 2*A*); we then conducted *in vitro* methylation reaction with recombinant NSUN2-GST protein expressed and purified *in vitro* (Fig 2*B*). The results show that full-length HBV pgRNA could be methylated by NSUN2, and its three parts had high methylation levels. Next, we used the *in vitro* bisulfite sequencing method to identify the sites of m^5^C modification (Fig 2*C*), during which the HBV genome was separated into 17 parts (200 nt each, Fig 2*A*) because the long fragment is unsuitable for this method. As shown in Fig 2*D* and S2 and S4 Tables, we found three sites with methylation levels above 50%: C131, C2017, and C2268 (relative to the unique single EcoRI site), and these three sites belong to fragments HBs3, HBe2, and HBe3, respectively. Site C131 is located at the open reading frame (ORF) of the S and P proteins, whereas sites C2017 and C2268 are located at the C protein ORF (Fig 2*E*). Other sites had methylation levels ranging from 10% to 40% (Fig 2*D* and S4 Table). Subsequently, we conducted the synonymous mutations of these sites on the fragments to which they belong (Fig 2*E*), except for C131 of which mutation is synonymous only for the polymerase protein but alters the amino acid sequence of the surface proteins. The results of *in vitro* methylation analysis show that the methylation level of these three fragments significantly decreased after mutation, which proved that these sites were the substrates of NSUN2 *in vitro* (Fig 2*F*). In the negative control, 100% of C nucleotides were converted to T nucleotides after bisulfite treatment, indicating that the conversion rate of *in vitro* sequencing met the sequencing requirements (S2 Fig).

**Fig 3:**
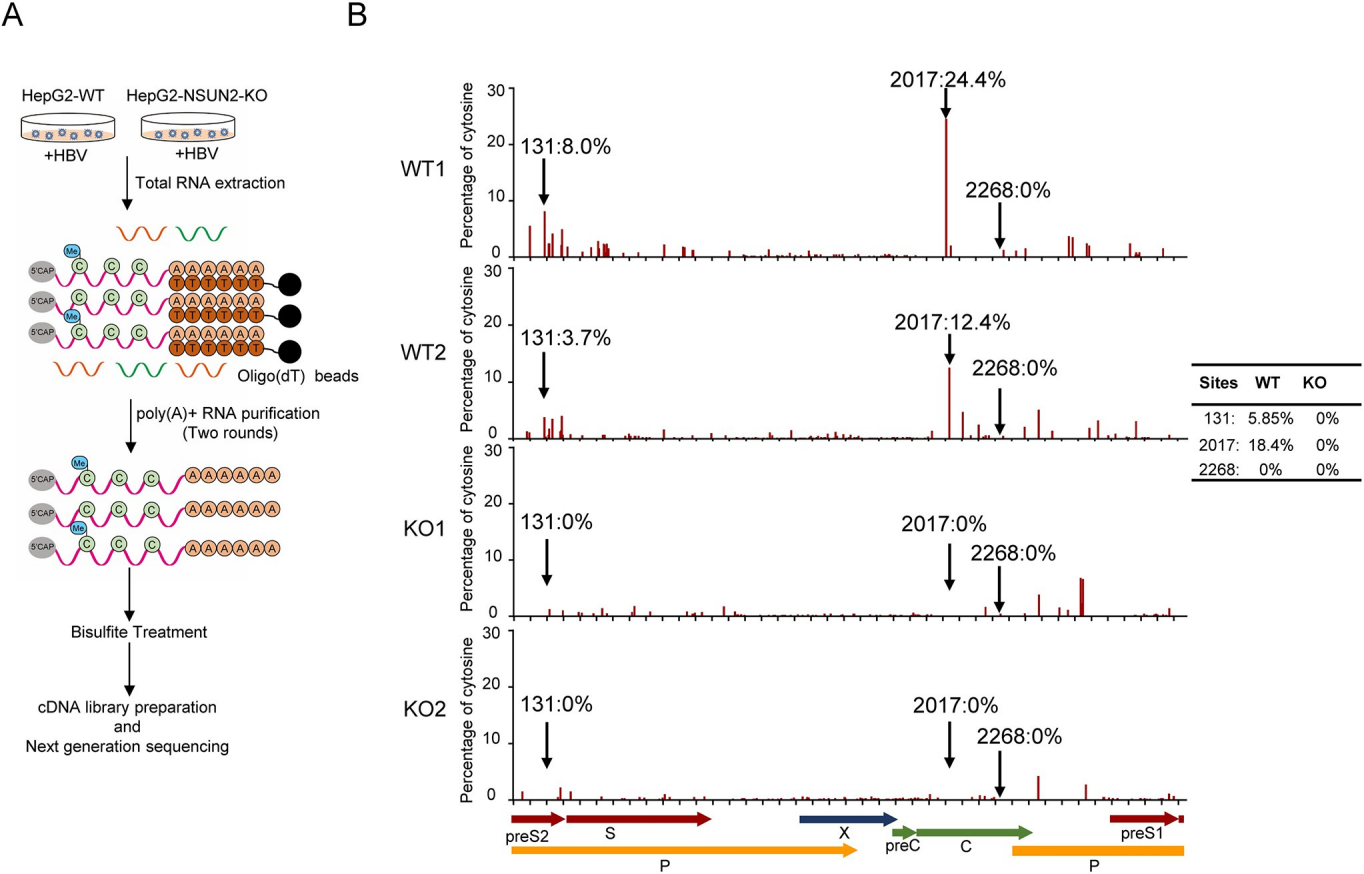
HBV RNA is methylated by NSUN2 in the cell. (*A*) Schematic diagram of high-throughput bisulfite sequencing. Total RNA from HepG2-WT or NSUN2-KO cells transfected with pHBV1.3 was subjected to poly-A purification for two rounds, followed by bisulfite treatment, cDNA library preparation, and next-generation sequencing. (*B*) Distribution and stoichiometry of m^5^C sites on HBV genome (NC_003977.2) from HepG2-WT and NSUN2-KO from two biological replicates. Sites C131, C2017, and C2268 are marked with black arrows. The y-axis shows the percentage of unconverted cytosines (m^5^C methylation rate), with a diagram of the viral genome shown at the bottom. The table in the top panel shows the average methylation rate of these sites.

To further determine the m^5^C methylation level of HBV RNA at the cellular level, we used high-throughput bisulfite sequencing with a combination of mRNA enrichment, bisulfite treatment, and next-generation sequencing (NGS) (Fig 3*A*). The cellular total RNA from HepG2 or HepG2 NSUN2-KO that were transfected with pHBV1.3 plasmid was prepared for sequencing. The results show that the m^5^C modification level in HBV RNA in the cell was lower than that for *in vitro* sequencing. Only one site, C2017, had the highest methylation rate of 18.4%, which became 0% after NSUN2 knockout. The methylation rate of site C131 was approximately 5.85%, which also became 0% after NSUN2 knockout. However, site C2268 was not methylated in the cell (Figs 3*B* and S3*A*), indicating that compared with *in vitro* sequencing, high-throughput sequencing is more reliable for reflecting the real methylation level, which may be due to the complicated regulatory mechanism in the cell. Nevertheless, *in vitro* transcribed and spiked-in luciferase RNA was used as the control, and its bisulfite conversion efficiency was 99.9%, indicating that the bisulfite treatment condition worked well in this sequencing process (Table 1). Taken together, these results confirm that HBV RNA is methylated by NSUN2 both *in vitro* and in the cell, with the highest methylation level at nucleotides C2017 and C131. In addition, when aligned with all known HBV genotypes from humans, sites C131 and C2017 are conserved, whereas site C2268 is not, which implies that sites with modifications might play important and conserved roles during the HBV life cycle (S3*B* Fig).

**Table 1:** Conversion rate of transcribed luciferase RNA spiked in high-throughput bisulfite sequencing.

	WT	KO
R1*	0.9989	0.9987
R2*	0.9990	0.9990

*: R1 and R2 means two biological replicates of high-throughput bisulfite sequencing; WT: HepG2-WT, KO: HepG2-NSUN2-KO.

### m^5^C modified sites C131 and C2017 contributes to HBV replication

**Fig 4:**
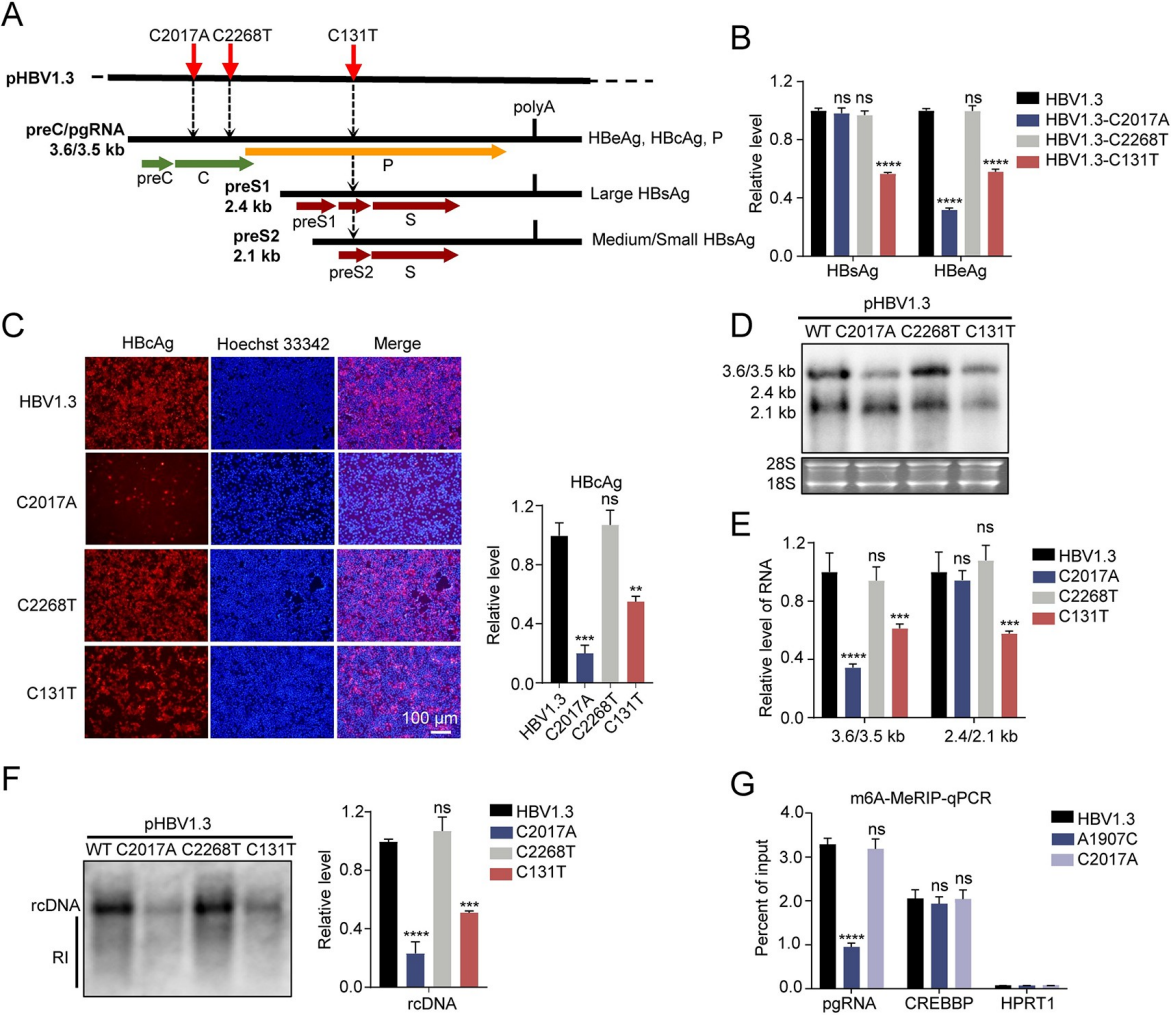
m^5^C modified sites C131 and C2017 positively regulate HBV replication. (*A*) Schematic diagram of site C2017, C2268, and C131 mutations on pHBV1.3. Red arrows indicate the position of these three sites. (B-F) pHBV1.3 and its mutants with pSV-β-gal were transfected into Huh7 cells. At 48 hours later, supernatant was collected for ELISA, and cells were harvested for lysis, RNA extraction, and rcDNA extraction. Activity of β-galactosidase in cell lysates was measured to normalize efficiency of transfection for ELISA. (*B*) ELISA results of pHBV1.3 and its mutants in Huh7 with β-galactosidase normalization. (*C*) Immunofluorescent staining (left) and fluorescence intensity analysis (right) of HBcAg in Huh7 after transfection with pHBV1.3 and its mutants. Relative levels were quantified using ImageJ. (*D*) Northern blot result of HBV RNA after transfection with pHBV1.3 and its mutants in Huh7. Ribosomal RNAs (28S and 18S) were used as loading controls. (*E*) Gray degree analysis of HBV RNA in Northern blot from Fig 4*D*. The relative levels of HBV RNA were quantified with Quantity One. (*F*) Southern blot (left) and gray degree analysis (right) of HBV core-associated rcDNA in cells after transfection with pHBV1.3 and its mutants. RI, replication intermediates. (*G*) MeRIP-qPCR of m^6^A methylated HBV RNA after C2017A and A1907C mutations in pHBV1.3. For A1907C mutation, both 5’ and 3’ DRACH motifs were mutated. *CREBBP* and *HPRT1*, were analyzed as m^6^A positive and m^6^A negative controls, respectively. Immunoblots shown are representative of three independent experiments. Graphs show the mean ± SD derived from three independent experiments and were analyzed by one-way ANOVA (one target) or two-way ANOVA analysis (two or more targets) followed by multiple comparisons test. ns, not significant for P > 0.05, *P < 0.05, **P < 0.01, ***P< 0.001, ****P< 0.0001.

To investigate the possible effects of these methylation sites on HBV RNA, we constructed the corresponding mutations on pHBV1.3 plasmid, which we named C2017A, C2268T, and C131T (Fig 4*A*). In Huh7 cell lines transfected with these mutant plasmids, the level of HBeAg significantly decreased after mutation of C2017A, but the level of HBsAg was not affected (Fig 4*B*). The HBsAg level notably decreased after C131T mutation, whereas the HBeAg level slightly decreased (Fig 4*B*). In addition, C2268T mutation had no noticeable effect on HBsAg and HBeAg expression (Fig 4*B*). Immunofluorescent staining of HBcAg showed that C2017A mutation resulted in a huge reduction in HBcAg expression, C131T mutation led to weak reduction, and C2268T mutation made no difference (Fig 4*C*). Then, we extracted the total RNA for Northern blot assay to investigate the effect of mutations on the RNA level of HBV (Fig 4*D* and 4*E*). The results showed that C2017A mutation remarkably degraded preC/pgRNA but had no noticeable effect on the preS1 and preS2/S RNA. In contrast, C131T mutation slightly degraded the preS1, preS2/S RNA, and preC/pgRNA. However, we observed no change in HBV RNA level after C2268T mutation (Fig 4*D* and 4*E*). Further detection of HBV core-associated rcDNA in cells revealed that both C2017A and C131T mutations resulted in its remarkable reduction, whereas C2268T had no effect (Fig 4*F*). To exclude the possibility of potential m^6^A modifications introduced by the C2017A mutation, we used m^6^A-MeRIP-qPCR to assess the levels of m^6^A modifications following C2017A mutation on pHBV1.3. The results did not reveal obvious change in m^6^A modification levels after C2017A mutation (Fig 4*G*), while A1907C mutation showed a marked decrease. A1907 on HBV RNA was reported to be methylated with m^6^A [17]. CREBBP and HPRT1 were analyzed as m^6^A positive and negative controls, respectively. These results indicate that m^5^C sites on HBV RNA, including sites C2017 and C131, can positively regulate HBV expression and replication.

### m^5^C modified sites contributes to HBV replication in cccDNA-like system

The aforementioned experiments were conducted using mutated plasmids, wherein viral RNA is transcribed from the plasmid. However, in actual HBV infection scenarios, viral transcripts are derived from the covalently closed circular DNA (cccDNA) transcription. To validate whether similar results can be observed in the cccDNA system after mutations, we employed the prcccDNA/pCMV-Cre system [41], which utilizes the Cre recombinase to generate a surrogate for cccDNA known as rcccDNA (S4*A* Fig), initiating viral transcription and replication. We evaluated the levels of viral transcripts derived from the rcccDNA or its mutants, as well as the subsequent antigen expression and DNA replication levels. The results demonstrated that C2017A mutation significantly degraded viral transcripts (3.6/3.5 kb) without affecting rcccDNA levels (S4*B* Fig). Moreover, it led to reduced levels of rcDNA and HBeAg (S4*B* and S4*C* Fig). In contrast, C2268T mutation had no discernible impact. These findings indicate that mutations of m^5^C modified sites influence HBV viral replication in the cccDNA-like system.

### NSUN2-mediated m^5^C modifications contributes to HBV RNA stability

**Fig 5:**
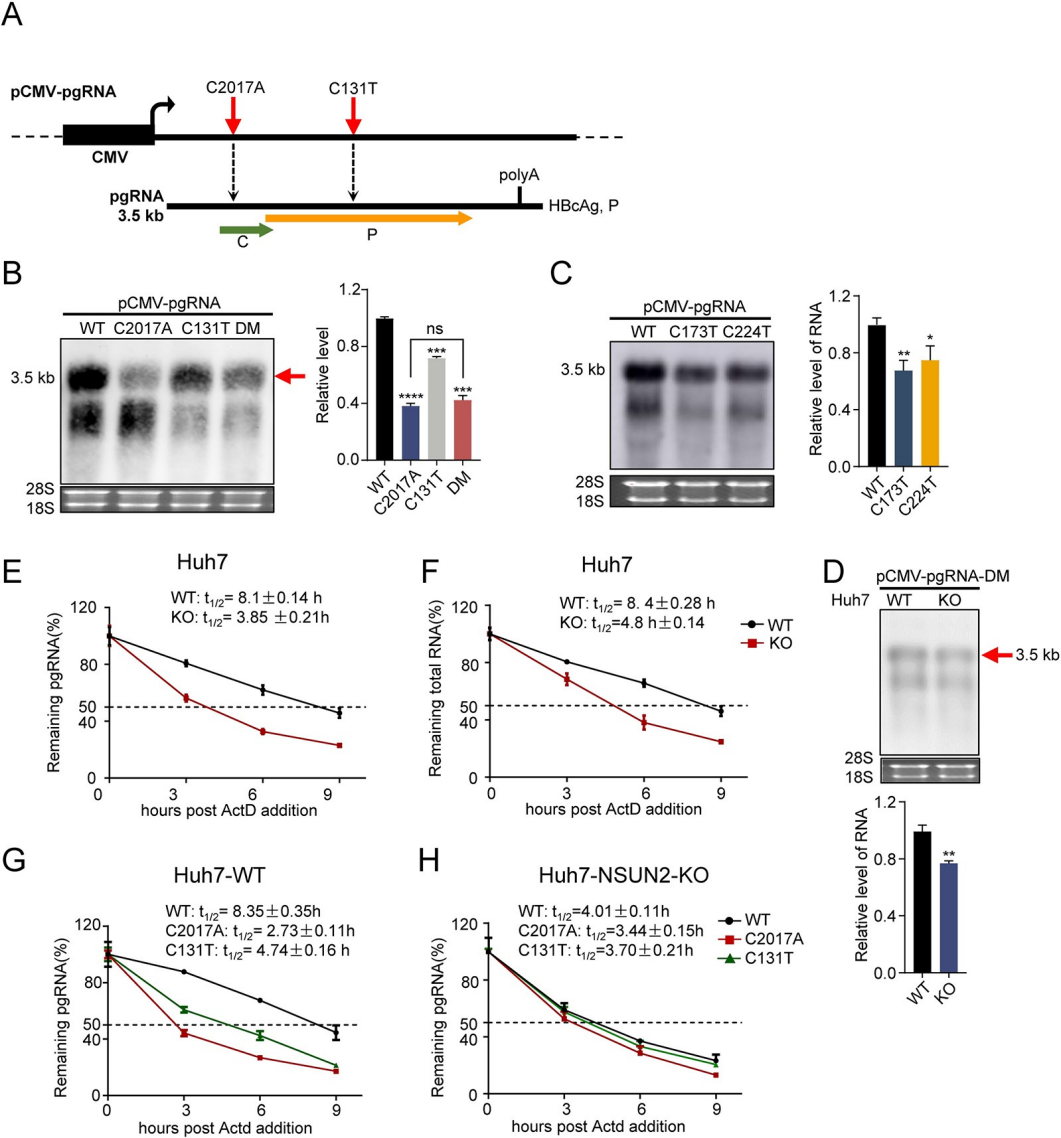
NSUN2-mediated m^5^C in HBV RNA contributes to HBV RNA stability. (*A*) Schematic diagram of site C2017 and C131 mutations on pCMV-pgRNA. Red arrows indicate positions of these three sites. (*B*) Northern blot result (left) and gray degree analysis (right) of HBV pgRNA after transfection with pCMV-pgRNA and its mutants, including C2017A, C131T, and double-mutation (DM). pCMV-pgRNA and its mutants were transfected into Huh7 cells. At 48 hours later, cells were harvested for RNA extraction. Ribosomal RNAs (28S and 18S) were used as loading controls. (*C*) Northern blot result (left) and gray degree analysis (right) of HBV RNA after transfection with pCMV-pgRNA and additional two mutants: C173T and C224T. (*D*) Northern blot result (top) and gray degree analysis (bottom) of HBV RNA after transfection with pCMV-pgRNA-C2017A&C131T (DM) in Huh7 with and without NSUN2 knockout. (*E* and *F*) Half-life of HBV preC/pgRNA and total RNA after NSUN2 knockout in Huh7. pHBV1.3 was transfected into Huh7 and Huh7-NSUN2-KO cells. At 36 hours later, actinomycin D (ActD, 15 ug/mL) was added, and then cells were harvested after 0, 3, 6, and 9 hours for qPCR with GAPDH as the control. (*G*) Half-life of HBV pgRNA from pCMV-pgRNA and its mutants in Huh7-WT. pCMV-pgRNA and its mutants were transfected into Huh7 cells. At 36 hours later, actinomycin D (ActD, 15 μg/mL) was added into the culture medium, then cells were harvested at 0, 3, 6, and 9 hours for RNA extraction and qPCR with GAPDH as the control. (*H*) Half-life of HBV pgRNA from pCMV-pgRNA and its mutants in Huh7-NSUN2-KO. Graphs show the mean ± SD derived from three independent experiments and were analyzed by one-way ANOVA (one target) or two-way ANOVA analysis (two or more targets) followed by multiple comparisons test. ns, not significant for P > 0.05, *P < 0.05, **P < 0.01, ***P< 0.001, ****P< 0.0001.

To exclude the possibility that the mutation of S protein might affect the preC/pgRNA level and the possibility that the mutations of plasmids expressing HBV might affect the transcription ability of viral promoters, the HBV pgRNA sequence and its corresponding mutants were constructed into the vector initiated by CMV promoter, named pCMV-pgRNA (Fig 5*A*). As shown in Fig 5*B*, compared with the wild-type pCMV-pgRNA, C2017A mutation led to a significant decrease in the RNA level, and C131T also slightly degraded pgRNA, while double mutations didn’t result in lower levels of pgRNA, suggesting the absence of a synergistic effect. Similarly, synonymous mutations of other two m^5^C methylated sites with low methylation level (~4%, C173T, C224T, S1 Table) also led to mild decreases of pgRNA levels compared with the wild type (Fig 5*C*). Furthermore, we compared the changes in HBV RNA levels with and without NSUN2 knock out by using the double-mutant pCMV-pgRNA plasmid (Fig 5*D*). The results showed that the levels of HBV RNA decreased slightly after NSUN2 knockout, suggesting that other m^5^C sites may also play a regulatory role. These results indicate that the pgRNA degradation caused by these site mutations is not related to transcription but post-transcriptional.

It has been reported that m^5^C modifications can regulate mRNA stability [27]. This prompts the question of whether NSUN2-mediated m^5^C modifications can also regulate the stability of HBV RNA. To address this question, the half-life of HBV preC/pgRNA RNA and total RNA after NSUN2 knockout was determined. The results showed that the preC/pgRNA half-life decreased from 8.1 h to 3.85 h after NSUN2 knockout, whereas that of total RNA decreased from 8.4 h to 4.8 h (Fig 5*E* and 5*F*), which proves that NSUN2 depletion led to the decline in HBV RNA stability. Similarly, when the expression of TET2 was depleted, the half-life of HBV preC/pgRNA increased from 7.98 h to 12.26 h, during which the level of m^5^C modification in pgRNA increased (S5*A* and S5*B* Fig). Furthermore, the results showed that the half-life of HBV pgRNA significantly decreased after C2017A mutation from approximately 8.35 h to 2.73 h and slightly decreased after C131T mutation from approximately 8.35 h to 4.74 h (Fig 5*G*). However, in the Huh7-NSUN2-KO cells, there were no significant differences in the half-life among them (Fig 5*H*). In addition, to specifically assess the effect of NSUN2-KO on HBV subgenomic RNAs, plasmids transcribing these subgenomic RNAs (2.4/2.1/0.7 kb) were transfected into Huh7, followed by Northern blotting to examine the changes after NSUN2 knockout. The results revealed a significant decrease in the levels of HBV subgenomic RNAs upon NSUN2 depletion (S5*C* Fig). In comparison, slighter change was observed in the 0.7 kb RNA relative to other subgenomic RNAs, which is consistent with high-throughput bisulfite sequencing results revealing that m^5^C modification levels on 0.7kb RNA were indeed lower (Fig 3*B*). Taken together, we conclude that m^5^C modifications in HBV RNAs mediated by NSUN2 play a key role in HBV post-transcriptional regulation by maintaining RNA stability.

### NSUN2 promotes HBV infection and replication in HepG2-NTCP and primary human hepatocytes

**Fig 6:**
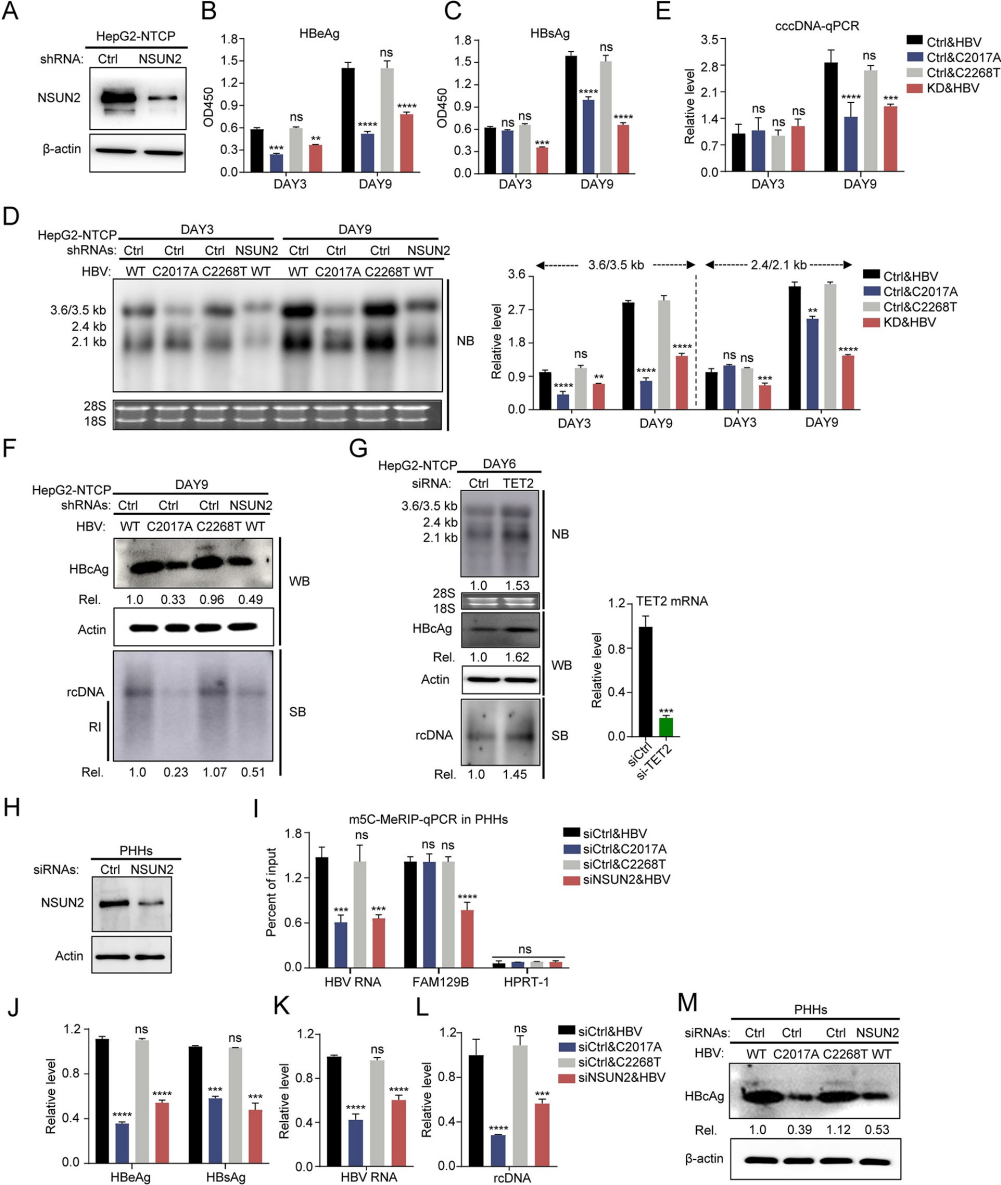
NSUN2 depletion negatively regulates HBV expression, replication, and infection in HepG2-NTCP or primary human hepatocytes infection system. (A-F) NSUN2 in HepG2**-**NTCP was depleted by using a lentivirus-delivered short hairpin RNA (shRNA). For HBV infection, HepG2-NTCP cells were plated into a 96-well plate. At 24 hours later, cells were infected by wild-type or mutated HBV with a multiplicity of infection (MOI) of 400. At 3 and 9 days after infection, supernatant was harvested for ELISA of HBV antigens, from which the OD values at a 450 nm wavelength were determined, and cells were harvested for RNA, rcDNA, cccDNA, and protein extraction. (*A*) Western blot of NSUN2 protein after NSUN2 depletion in HepG2**-**NTCP. (*B* and *C*) ELISA results of HBV antigens on days 3 and 9 after infection. (*D*) Northern blot result (left) and gray degree analysis (right) of HBV RNA on days 3 and 9 after infection. (*E*) HBV cccDNA-qPCR results in HepG2**-**NTCP on days 3 and 9 after infection. (*F*) Western blot result (top) of HBcAg and Southern blot (bottom) result of HBV rcDNA on days 9 after infection. RI, replication intermediates. (*G*) Northern blot result of HBV RNA (top), Western blot result (medium) of HBcAg and Southern blot (bottom) result of HBV rcDNA on days 6 after infection. HepG2-NTCP cells were plated into a 96-well plate and transfected with siRNA for TET2 depletion. At 24 hours later, cells were infected by wild-type HBV with a multiplicity of infection (MOI) of 400. TET2 mRNA level (right) after depletion was determined with qPCR. RI, replication intermediates. (H-M) Primary human hepatocytes were plated into 96-well plates and transfected with siRNA targeting NSUN2. At 24 hours later, PHHs were infected by wild-type or mutated virus. 9 days after infection, supernatant was harvested for ELISA, and cells were harvested for RNA, rcDNA, and protein extraction, then subjected for qPCR or Western blot. (*H*) Western blot of NSUN2 protein after NSUN2 depletion in PHHs. (*I*) MeRIP-qPCR of m^5^C methylated HBV transcripts in PHHs after infection. FAM129b and HPRT1 serve as m^5^C positive and negative controls, respectively. (*J*) ELISA results of HBV antigens on 9 days after infection. (*K* and *L*) HBV RNA and rcDNA qPCR results on 9 days after infection. (*M*) Western blot results of HBcAg on 9 days after infection. Immunoblots shown are representative of three independent experiments. Graphs show the mean ± SD derived from three independent experiments and were analyzed by one-way ANOVA (one target) or two-way ANOVA analysis (two or more targets) followed by multiple comparisons test. ns, not significant for P > 0.05, *P < 0.05, **P < 0.01, ***P< 0.001, ****P< 0.0001.

To investigate whether these results could be repeated in the HBV infection system, we analyzed the HBV expression and replication levels in NSUN2-depleted HepG2-NTCP after infection with HBV. Depletion of NSUN2 in HepG2-NTCP is mediated by shRNA-expressing lentivirus infection (Fig 6*A*). HBV virus, including wild type, C2017A mutant, and C2268T mutant, were produced by transfection into Huh7. The C131T mutant with a changed amino acid sequence was not produced. The results show that the antigens and RNAs levels of HBV decreased after NSUN2 depletion at 3 and 9 days post-infection (Fig 6*B*, *6C,* and 6*D*). In addition, compared with the wild-type virus, C2017A mutation significantly reduced HBeAg and 3.6/3.5 kb RNA levels at 3 and 9 days post-infection (Fig 6*B* and 6*D*). However, at 9 days post-infection, C2017A mutation also reduced HBsAg and 2.4/2.1 kb RNA levels which were not affected on day 3 (Fig 6*C* and 6*D*). To explain the inconsistency, the levels of cccDNA were determined with qPCR, and results showed that there were no significant differences in cccDNA levels among the groups on day 3 (Fig 6*E*). However, on day 9, both mutation and NSUN2 knockdown led to a significant decrease in cccDNA levels. These results imply that mutation and NSUN2 knockdown result in reduced progeny virus production in the early stage of viral infection, consequently leading to a decrease in replenished cccDNA levels in the late stage. Subsequently, the HBcAg and rcDNA levels were analyzed at 9 days post-infection and show significant decline after C2017A mutation or NSUN2 knockdown, while C2268T mutation made no difference (Fig 6*F*). In addition, when the expression of TET2 in HepG2-NTCP was depleted, HBV RNAs, HBcAg, and rcDNA levels increased at 6 days post-infection (Fig 6*G*).

We further validated these results in primary human hepatocytes (PHHs). PHHs were infected with wild type, C2017A mutant, or C2268T mutant HBV virus, after siRNAs transfection targeting NSUN2 or control (Fig 6*H*). At 9 days post-infection, HBV RNAs from infected PHHs were subjected to methylated RNA immunoprecipitation (MeRIP) assay with an m^5^C-specific antibody followed by RT-qPCR using primers that specifically recognize HBV pgRNA (Fig 6*I*). The results showed that both C2017A mutation and NSUN2 knockdown in PHHs led to significant reduction of m^5^C methylation in HBV pgRNA, which are consistent with previous findings. In the MeRIP assay, FAM129B, a cellular RNA known to contain m^5^C and HPRT1, a cellular RNA that does not contain m^5^C, were used as positive and negative controls, respectively [9, 31]. Subsequently, HBV antigens, RNAs, and rcDNA levels were also analyzed with ELISA, qPCR, and Western blotting for HBcAg and show remarkable decrease after C2017A mutation and NSUN2 knockdown (Fig 6*J*, 6*K*, 6*L*, and *6M*). Based on these results, we conclude that NSUN2-mediated m^5^C modification on HBV RNA could also positively regulate HBV infection and replication in HepG2-NTCP and primary human hepatocytes.

### HBV expression and HBV core protein positively regulate the expression of endogenous NSUN2

**Fig 7:**
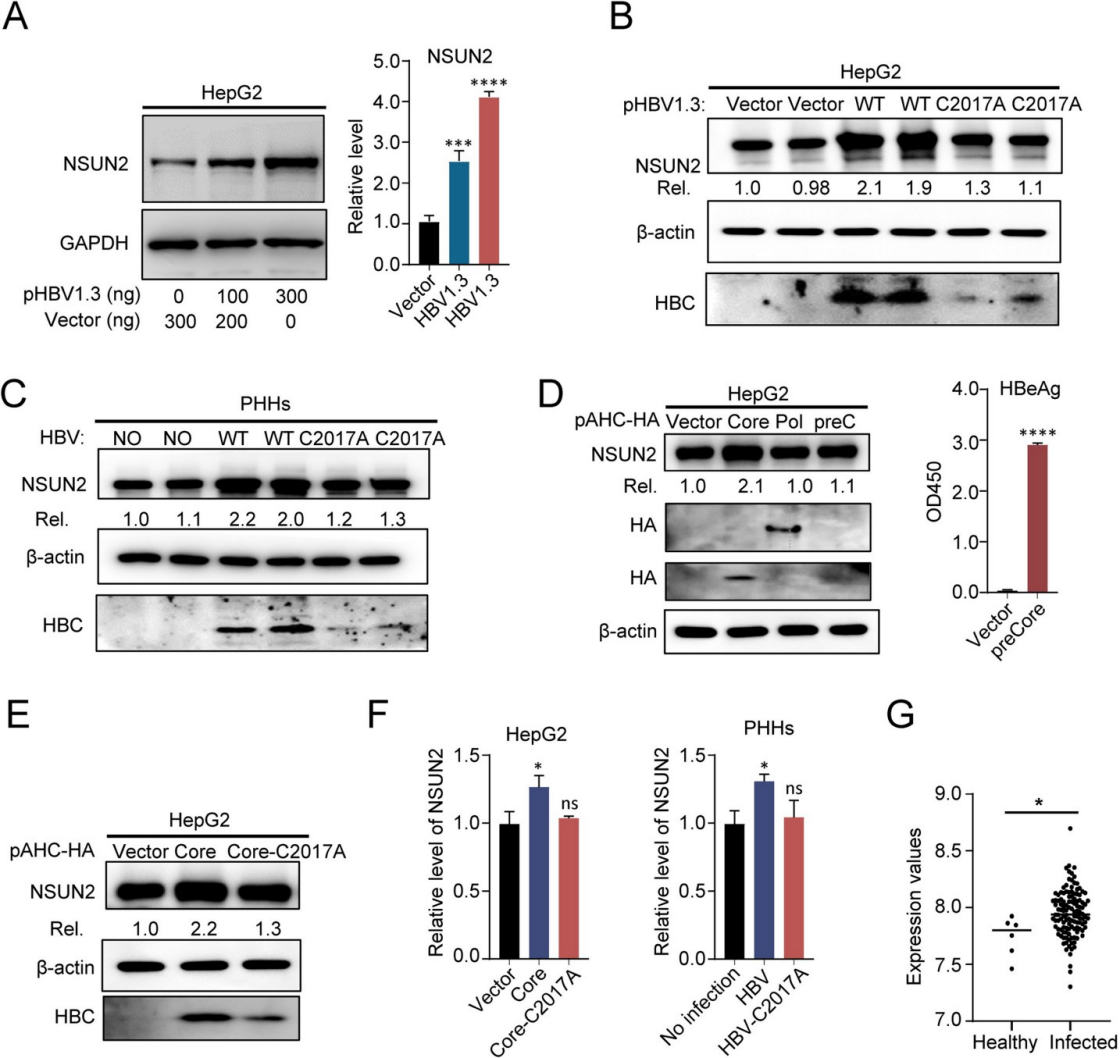
HBV expression positively regulates expression of endogenous NSUN2. (*A*) Western blot (left) and gray degree analysis (right) of endogenous NSUN2 in HepG2 after pHBV1.3 gradient transfection. Relative levels of NSUN2 protein to GAPDH were quantified using Quantity One. At 48 hours after pHBV1.3 transfection, cells were harvested for Western blot. (*B*) Western blot of endogenous NSUN2 in HepG2 after transfection with pHBV1.3 and its mutant. Relative levels of NSUN2 protein were quantified using Quantity One. (*C*) Western blot (left) of endogenous NSUN2 after HBV infection in PHHs with wild-type or mutated virus. At 9 days after HBV infection, cells were harvested for Western blot. The relative levels of NSUN2 protein were quantified using Quantity One. (*D*) Western blot of endogenous NSUN2 in HepG2 after transfection with pAHC-Core, pAHC-Pol, and pAHC-preC. The expression of HBV Pol and Core were analyzed with antibody against HA. The expression of HBeAg was analyzed with ELISA. (*E*) Western blot of endogenous NSUN2 in HepG2 after transfection with pAHC-Core and pAHC-Core-C2017A. (*F*) qPCR results of NSUN2 mRNA levels after core protein transfection in HepG2 or infection in PHHs. GAPDH was used as the internal reference. (*G*) NSUN2 expression level in a published dataset from healthy or HBV infected human liver tissue (GEO: GSE83148). Immunoblots shown are representative of three independent experiments. Graphs show the mean ± SD derived from three independent experiments and were analyzed by one-way ANOVA analysis or unpaired Student’s t test. ns, not significant for P > 0.05, *P < 0.05, ***P< 0.001, ****P< 0.0001.

To investigate whether HBV expression could influence endogenous NSUN2 expression, the expression of NSUN2 after gradient transfection of pHBV1.3 into HepG2 was analyzed. The results show that NSUN2 expression increased with gradient transfection of pHBV1.3 compared with that of the vector group (Fig 7*A*). However, when transfected with C2017A mutant, the expression of endogenous NSUN2 only showed slight increase in HepG2 (Fig 7*B*). Similar results were also found in PHHs when infected with WT or mutated virus (Fig 7*C*). Collectively, these results suggest that HBV expression or replication can also positively regulate the expression of endogenous NSUN2 in turn.

Furthermore, the observed phenomena following C2017A mutation prompts us to speculate whether the proteins of which expression could be affected by C2017A mutation may serve as a key regulator of NSUN2. Based on this hypothesis, we separately constructed the regions encoding viral proteins including the core, polymerase (Pol), and precore (preC) into the expressing vector, and subsequently transfected them into HepG2 cells, since the expression of these three proteins could be affected by C2017A mutation. The results showed that the core protein significantly enhanced the expression of NSUN2 after transfection, while the Pol or preC proteins had no effect (Fig 7*D*). The expression of the HBeAg protein was checked with ELISA. Additionally, when C2017A mutation was introduced solely into the core expressing plasmid, we observed a slight increase in endogenous NSUN2 expression after transfection, which is consistent with the results after viral transfection or infection (Fig 7*E*). Subsequently, we investigated the impact of the core protein transfection and viral infection on NSUN2 mRNA levels. The results revealed that both viral infection and the core protein transfection led to a modest increase in NSUN2 mRNA level (Fig 7*F*).

Based on these results, we conclude that the HBV core protein may serve as a key regulator of endogenous NSUN2 expression. Moreover, HBV virus or the core protein likely exerts its enhancing effects by increasing NSUN2 mRNA levels. However, it is also plausible that alternative regulatory mechanisms are involved in this process. We also analyzed a published dataset from human liver tissue (GEO: GSE83148) and found that there was slight increase in hepatic NSUN2 expression in HBV infected samples (Fig 7*G*) [42]. This finding provides further clinical implications for our conclusions regarding the host-virus interaction.

### NSUN2 positively regulates the replication of HBV *in vivo*

**Fig 8:**
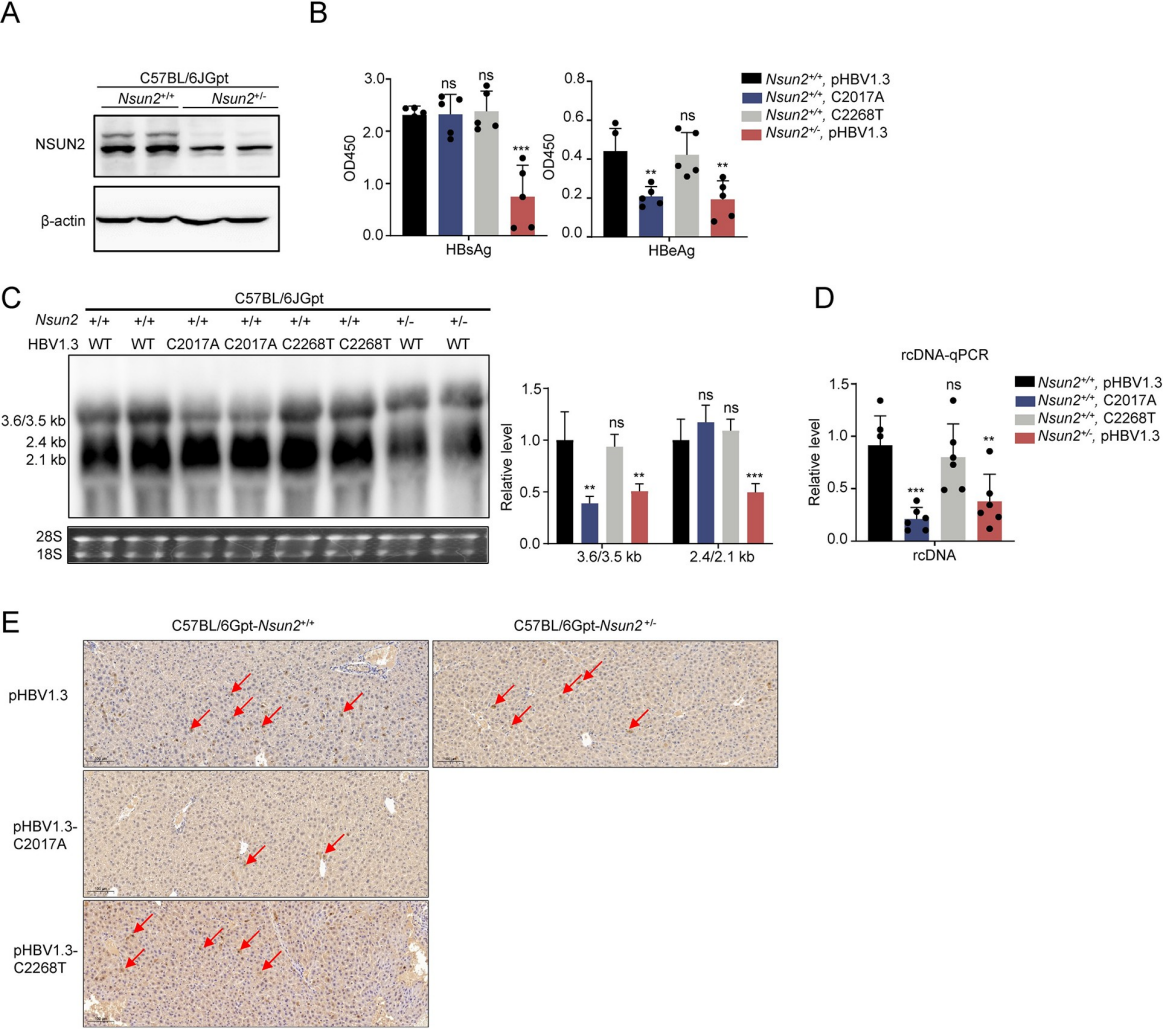
NSUN2 positively regulates HBV expression and replication *in vivo*. (A-F) Three groups of C57BL/6JGpt-*Nsun2*^+/+^ mice (six in each group) and one group of C57BL/6JGpt-*Nsun2*^+/-^ mice were prepared for *in vivo* experiments. Plasmids including pHBV1.3, pHBV1.3-C2017A, and pHBV1.3-C2268T were delivered into mice with hydrodynamic injection. 4 days after injection, mice blood and liver tissues were harvested for analysis. (*A*) Western blot of NSUN2 protein in *Nsun2*^+/+^ and *Nsun2*^+/-^ mice. (*B*) HBV antigens including HBsAg and HBeAg were detected with ELISA from mice sera. (*C*) Northern blot (left) and gray degree analysis (right) of HBV RNA from mice liver. (*D*) HBV rcDNA qPCR results in mouse sera. (*E*) Immunohistochemical staining of HBcAg from mice liver. The red arrow indicates the expression of HBcAg. Graphs show the mean ± SD derived from three independent experiments and were analyzed by one-way ANOVA analysis. ns, not significant for P > 0.05, **P < 0.01, ***P< 0.001, ****P< 0.0001.

To further validate the aforementioned conclusions *in vivo*, C57BL/6Gpt-*Nsun2*^+/-^ mice were generated and used for experiments after genotyping (S6*A*–S6*D* Fig). According to all the genotyping results, no homozygote mice was detected. And Western blotting analysis revealed a near 50% reduction of NSUN2 expression levels in *Nsun2*^+/-^ mice compared with *Nsun2*^+/+^ mice (Fig 8*A*). Subsequently, pHBV1.3, pHBV1.3-C2017A, and pHBV1.3-C2268T plasmids were delivered into wild-type or NSUN2 knock-down mice through hydrodynamic injection. At 4 days post-injection, the mice were euthanized, and blood and liver tissues were collected for ELISA, RNA, rcDNA extraction, and immunohistochemical analysis. The results showed that both C2017A mutation and NSUN2 knockdown led to a significant reduction in HBV antigens, RNA, and rcDNA levels (Fig 8*B*, 8*C*, and 8*D*). Immunohistochemical analysis also revealed a consistent trend wherein the expression levels of core protein decreased due to NSUN2 knockdown or C2017A mutation (Fig 8*E*). Taken together, we conclude that NSUN2 positively regulates HBV replication *in vivo*.

## Discussion

**Fig 9:**
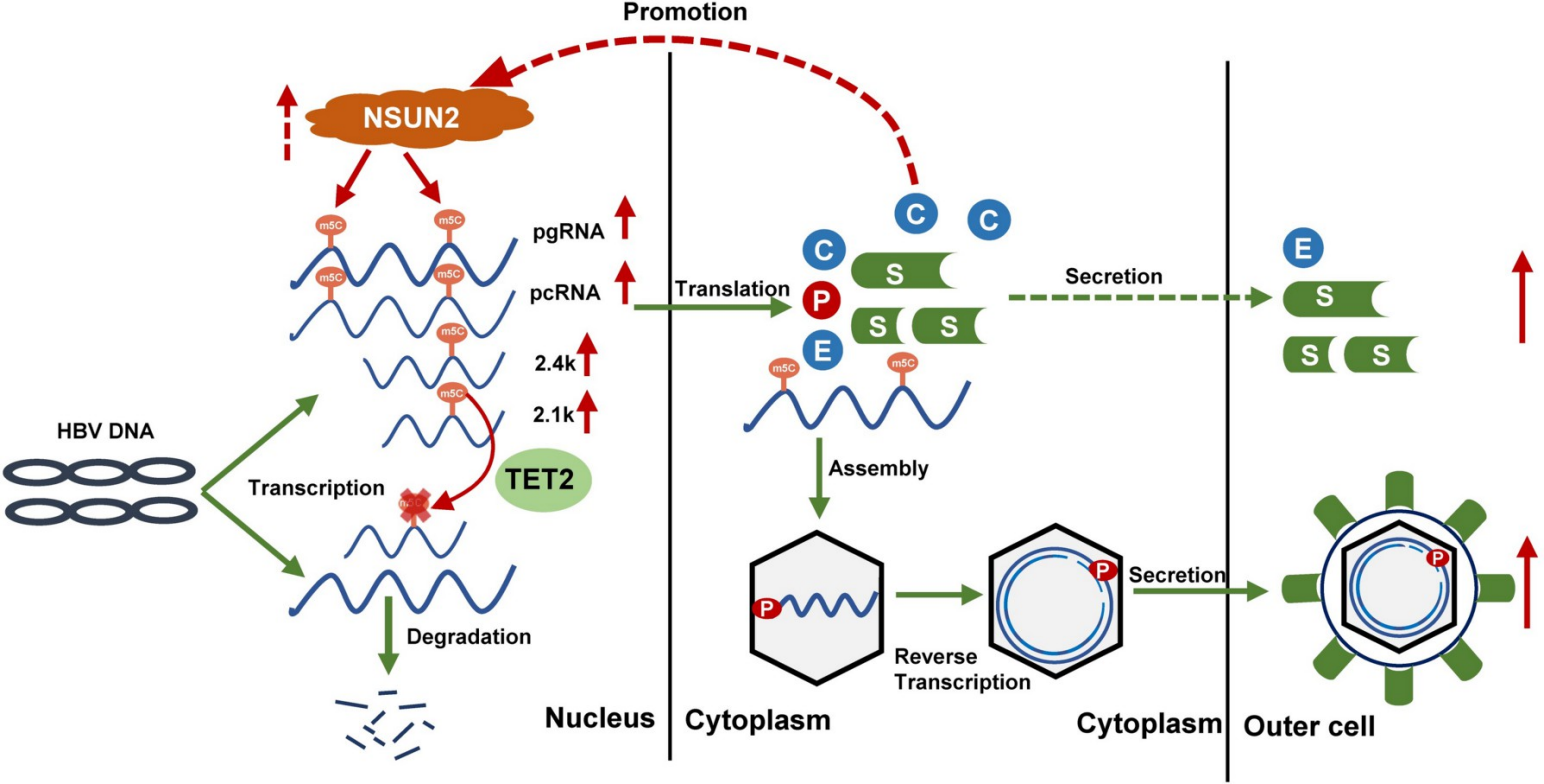
Schematic diagram of regulation between NSUN2 and HBV. NSUN2-mediated m^5^C methylation positively regulates expression and replication of HBV during which TET2 plays the opposite role with demethylation; in turn, HBV expression or HBcAg affects endogenous NSUN2 expression.

Chronic hepatitis B infection caused by HBV still presents a challenging threat to human life and health. Although drugs are being developed to target different parts of the life cycle of HBV, the complete elimination of HBV is not yet possible. With the in-depth study of the replication mechanism behind HBV infection, we are advancing toward the achievement of this goal, including the study of epigenetic regulation of HBV RNA [17]. In this study, we found that NSUN2 depletion by knockdown or knockout resulted in decreased expression and replication of HBV, while TET2 deficiency positively regulates the expression of HBV. Subsequently, we demonstrated the presence of m^5^C modification on HBV RNA at single-nucleotide resolution with *in vitro* and high-throughput bisulfite sequencing methods, including for sites C2017 and C131, which are NSUN2-dependent and evolutionarily conserved. Mutations of these m^5^C sites significantly impair viral replication. Mechanistically, m^5^C modifications mediated by NSUN2 promote the stability of HBV RNA. Similar results were also obtained in the HepG2-NTCP and primary human hepatocytes infection system. Furthermore, we performed *in vivo* validation experiments using C57BL/6JGpt-*Nsun*2^+/-^ mice and came to the same conclusion. Together, these results suggest that m^5^C modification in HBV RNA mediated by NSUN2 has a positive regulatory effect on the HBV life cycle (Fig 9).

Currently, fewer studies have been conducted on the m^5^C modification of viruses than those on m^6^A modification, partly because of the low-level distribution of m^5^C and partly because the treatment of bisulfite in the sequencing process leads to rigorous RNA degradation. Therefore, effectively identifying m^5^C sites on RNA with low abundance or a low methylation level is difficult [43, 44]. However, researchers have also used *in vitro* m^5^C sequencing method to avoid these problems [45, 46]. In this study, we first combined these two methods to analyze the distribution of m^5^C methylation on HBV RNA (Figs 2 and 3). We identified three sites, i.e., C2017, C2268, and C131, with methylation levels above 50%, using an *in vitro* sequencing method (Fig 2*D*). We also identified two sites, C2017 and C131, with methylation levels of 18.4% and 5.6%, respectively, using a high-throughput sequencing method, whereas site C2268 was not methylated (Fig 3*B*). Subsequent functional mutations showed that site C2268 had no effect (Fig 4). The results show that *in vitro* sequencing was able to reveal the most direct interaction between methyltransferase and its substrate without the involvement of other regulatory proteins, as an overestimated methylation level and “false” methylated sites were obtained. However, high-throughput sequencing is more accurate for revealing the real methylation situation with lower methylation levels, in which unknown accessory proteins might participate. As a result, considering the lower threshold, we suggest that *in vitro* sequencing is more suitable for preliminary experiments exploring the direct interaction between RNA and methyltransferase.

To investigate the possible role of m^5^C methylated sites on HBV life cycles, we introduced mutations into HBV expressing plasmids, including pHBV1.3, prcccDNA/pCMV-Cre system, and pCMV-pgRNA, and then analyzed HBV RNAs and antigens levels after mutations. The results showed that mutations of these methylated sites led to decreased HBV RNAs and antigens levels through post-transcriptional regulation instead of affecting the level of transcription template (rcccDNA) or the transcriptional activity of HBV promoters (S4 and S5 Figs). Furthermore, m^5^C modification of viral RNA involves several main functions: splicing, trafficking, translation, and stability. In this study, we found that m^5^C could maintain the stability of HBV RNA (Fig 5), which is consistent with the findings of most studies on cellular mRNA or HIV-1 RNA [24, 26, 27, 47]. However, one study reported that m^5^C modification of Epstein–Barr virus (EBV) noncoding RNA led to RNA degradation [37]. This may be an isolated case, as this degradation process is mediated by RNase angiogenin (ANG), and its cleavage ability was reported to be counteracted by m^5^C in tRNA instead of facilitation [48]. Moreover, we found an interesting phenomenon: mutation C2017A led to considerable degradation of HBV preC/pgRNA, whereas mutation C131T led to partial degradation of preC/pgRNA (Fig 5*B*). Combined with the methylation levels and effects of these two sites (Fig 3*B*), we speculate that the effect of m^5^C sites on RNA is related to the methylation level and a higher level of methylation corresponds to a stronger binding affinity of reader proteins and increased stability. Besides, other m^5^C methylated sites, such as C173 and C224 (S1 Table and Fig 5*C*) with low levels obtained from high-throughput bisulfite sequencing also played similar roles as C2017 or C131. Taken together, all these methylation sites are involved in regulating the HBV life cycle. Furthermore, a recent study reported that loss of NSUN2 affects the cellular transcriptome and m^5^C epitranscriptome, as well as the cell viability [49]. Based on this report and our results in Fig 5*D*, we speculate that NSUN2 deficiency may also indirectly affect HBV RNA levels. However, it is important to note that other unmutated m^5^C methylation sites may also play a regulatory role on HBV RNA, even at low levels, as shown in Fig 5*C*. Therefore, it is challenging to accurately assess the indirect effect. Nevertheless, the conclusions of this study fully support a direct regulatory role of m^5^C modification in HBV RNA mediated by NSUN2.

In addition to the transfection system mentioned above, we also conducted validation experiments in the context of HBV infection, particularly in primary human hepatocytes (Fig 6). The results showed that HBV RNAs were methylated with m^5^C upon HBV infection in primary hepatocytes. Moreover, both NSUN2 knockdown and site mutation attenuated viral replication in PHHs. Furthermore, we also performed *in vivo* validation experiments using C57BL/6JGpt-*Nsun*2^+/-^ mice with depleted expression of NSUN2 (Fig 8). We conclude that NSUN2-mediated m^5^C methylation positively regulates HBV replication in both PHHs and mice. These findings further support the physiological relevance of our study and provide important insights into the role of m^5^C modifications on HBV replication across different biological systems.

Interestingly, we found that HBV expression, in turn, also influenced the expression of NSUN2. The endogenous expression of NSUN2 in HepG2 and PHHs increased after HBV transfection or infection, during which a positive feedback loop formed to promote HBV replication and expression (Fig 7). Further results found that the core protein may serve as a key regulator of endogenous NSUN2 expression and likely exerts its enhancing effect by increasing NSUN2 mRNA level. A published dataset from human liver tissue (GEO: GSE83148) was analyzed and showed that there was slight increase in hepatic NSUN2 expression in HBV infected samples. Similarly, Song *et al*. reported that NSUN2 was highly expressed in hepatocellular carcinoma (HCC) tissues, and the mRNA m^5^C modification in HCC tissues was also higher than that in the adjacent normal tissues [29, 50]. The hypermethylated target genes (*GRB2*, *AATF*, and *RNF115*), with increased expression, participate in carcinogenic pathways. However, whether HBV is involved in the formation of these HCCs was not disclosed, but the finding implies that HBV infection might promote hepatocarcinogenesis through promoting NSUN2 expression. Further research would be valuable in this respect.

Finally, two studies have reported that Y box binding protein 1 (YBX1) is a recognition protein of m^5^C-methylated RNA and can stabilize mRNA. Surprisingly, no significant changes were detected in the levels of HBV antigens or RNAs after the depletion of YBX1 (Fig 1*K* and 1*L*). This intriguing finding suggests the involvement of unidentified m^5^C reader proteins that may contribute to the regulation of HBV replication. Identifying and investigating the function of new m^5^C reader proteins regulating HBV RNA is the direction of our future research, which represents a significant extension of our findings in this manuscript.

In conclusion, our study provides the single-base-resolution RNA m^5^C landscape of HBV and reveals a functional model of m^5^C modification in the HBV life cycle, which may bear relevance to associated pathological syndromes. This study also provides a basis for the subsequent investigation of the detailed mechanism in which m^5^C recognition protein of HBV RNA might participate, and potential targets for the development of antiviral drugs.

## Materials and methods

### Ethics statement

All the animal experiments were performed according to the Regulations of Hubei Province Laboratory Animal Management and approved by Wuhan University Animal Experiment Ethics Committee under project licence WP20220100.

### Cell culture and plasmid construction

HepG2, Huh7, 293T, and HepG2-NTCP in this study were cultured in Dulbecco’s modified Eagle’s medium (DMEM, Gibco) with 10% fetal bovine serum, 100 U/mL penicillin, and 100 μg/mL streptomycin at 37°C in a 5% CO_2_ incubator. All the cells were tested for mycoplasma to exclude contamination. Primary human hepatocytes (PHHs) were obtained from Liver Biotechnology (China) and cultured according to the manufacturer’s protocol. The regions encoding HBV proteins including the core and polymerase were constructed into pAHC-HA vector as previously described and the plasmid encoding preCore was also constructed based on the same vector and method [51]. The sequence of primers used for constructing mutations of plasmid HBV1.3 is shown in S2 Table. The method for the construction of pCMV-pgRNA (3.5k), pCMV-2.4k, pCMV-2.1k, and pCMV-0.7k plasmids were previously described [52]. The prcccDNA/pCMV-Cre system was generously donated by Professor Deng of Fudan University [41]. Plasmid transfection was performed with Lipofectamine 3000 (Thermo Fisher). siRNAs targeting NSUN2, DNMT2, METTL3/14, YBX1, ALYREF, and TET2 were purchased from Ribobio Company (China) and transfected with LipoRNiMAX (Thermo Fisher) according to the instructions.

### Construction of NSUN2-knockout cell lines

Lentiv2-Crispr/Cas9-gNSUN2 plasmids were constructed as previously described [39], with the gRNA sequence provided in S2 Table. We transfected 293T cells with pMD2.G, psPAX2, and Lentiv2-Crispr/Cas9-gNSUN2 plasmids to produce lentivirus targeting endogenous NSUN2. The supernatants containing lentivirus were collected 48 h and 72 h post-transfection and purified with 0.22 μm filter membrane, then aliquoted and stored at -80°C. Huh7 or HepG2 cells were infected with lentivirus twice, for 4 hours each, with an interval of 24 h. Then, after multiple passages and puromycin selection, the cells were seeded into 96-well plates. Then, single-cell clones were selected and checked with Western blotting to obtain NSUN2-knockout cell lines.

### Short hairpin RNA (shRNA)-mediated gene silencing

The sequence of shRNAs specific to NSUN2 and control were listed in S2 Table. The shRNAs were cloned into pLKO.1 using EcoRI (ThermoFisher) and AgeI (ThermoFisher) and packaged into lentiviruses using psPAX2 and pMD2.G. Stable knockdown cell lines were screened by puromycin after lentiviral infection.

### Recombinant protein expression and in vitro transcription

The recombinant NSUN2-GST protein was expressed *in vitro* using pGEX-6p-1-GST-NSUN2, which was transformed into *Escherichia coli* BL21 cells and purified using gravity columns containing glutathione resin (GenScript, China). The purified recombinant proteins were aliquoted and stored in elution buffer (50 mM Tris-HCL, pH 8.0, 2 mM DTT, 10 mM reduced glutathione, and 8% glycerol) at -80°C until use.

Primers with T7 promoter and adaptor were used to amplify different regions of plasmid HBV1.3 (S3 Table), and then PCR products were used as templates for *in vitro* transcription with a HiScribe T7 High-Yield RNA Synthesis Kit (New England Biolabs). After 16 hours in a water bath at 30°C, DNase I (Invitrogen) was added to digest the DNA templates. The transcribed RNA was purified with ethanol precipitation and dissolved in RNase-free water, and checked with agarose gel electrophoresis.

### *In vitro* methylation assays

Reaction mixtures were prepared with reaction buffer (500 mM Tris-HCl, pH 7.5, 5 mM EDTA, 10 mM dithiothreitol, and 20 mM MgCl_2_), 0.2 nM recombinant NSUN2-GST, 0.01 nM *in vitro* transcribed RNA, 1 μCi of S-adenosyl [methyl-3H] methionine (SAM, 0.5 μCi/μL; PerkinElmer), and 40 units of RNase inhibitor, then incubated at 37°C for 60 min, as described previously [23]. The ^3^H-labeled products were purified using DEAE-Sephadex A-50 columns and quantitated with a liquid scintillation counting machine (PerkinElmer). No-isotopic methylated RNA fragments were similarly prepared, except using cold SAM (New England Biolabs). miR-125b mimic was used as the positive control [53]; the siRNA of the control was used as the negative control.

### *In vitro* bisulfite sequencing

*In vitro* transcribed RNA was methylated with cold SAM as above mentioned, then purified for bisulfite conversion with an EZ RNA Methylation Kit (Zymo research) according to the instructions: 70°C for 5 minutes and 64°C for 45 minutes, with 3 cycles. The converted RNA was reverse-transcribed with adaptor R primers and then amplified with adaptor primers. The amplified products were connected to the T vector, the positive clones were selected through blue-white spot screening, and then sequenced with Sanger sequencing in Sangong (China). The sequencing results were analyzed with DNMAN (version 7.0). The methylation rate of single C was calculated as the ratio of the reads number of unconverted C to the total number of sequenced reads.

### High-throughput bisulfite sequencing

pHBV1.3 was transfected into HepG2 and HepG2-NSUN2-KO cells. After 48 hours, TRIzol reagent was added to extract total RNA, and oligo-dT magnetic beads (New England Biolabs) were used to enrich mRNA with two cycles. The enriched 1 μg mRNA with 5 ng of luciferase RNA transcribed *in vitro* was subjected for bisulfite conversion using an EZ RNA Methylation Kit (Zymo research) according to the instructions: 70°C for 5 minutes and 64°C for 45 minutes, with 3 cycles. After purification, the converted mRNA was used for library construction using a KAPA RNA HyperPrep kit (Illumina). Sequencing was then performed using the Illumina platform of ABlife company (China). The sequencing data were analyzed with meRanTK (version 1.2.0) after quality control to identify the methylation distribution and stoichiometry of m^5^C sites on the HBV genome, and the conversion rate of luciferase control was analyzed. The raw sequencing data has been deposited in the Genome Sequence Archive (GSA) in National Genomics Data Center, China National Center for Bioinformation [54, 55], under the accession number HRA003981 that is publicly accessible at http://ngdc.cncb.ac.cn/gsa-human.

### ELISA for secretory hepatitis B antigens

HepG2 or Huh7 cells were cotransfected with pHBV1.3 and pSV-β-gal. After 48 h, the supernatant was collected. The levels of HBsAg and HBeAg antigens in the culture supernatant were assessed using an enzyme-linked immunosorbent assay kit (ELISA, Kehua Biotech). The OD values at a 450 nm wavelength were determined with a Multifunction microplate reader (Molecular Devices) according to the manufacturer’s protocol. Cells were lysed with passive lysis buffer (Promega). The activity of β-galactosidase in cell lysates was measured with a Beta-Glo kit (Promega) to normalize the efficiency of transfection.

### HBV core-associated rcDNA extraction and analysis

The process of extracting HBV rcDNA from intracellular core particles was performed as previously described with a few modifications [56]. In brief, HepG2 or Huh7 cells were transfected with pHBV1.3. At 48 h post-transfection, the cells were lysed in NP-40 lysis buffer (50 mM Tris-HCl (pH 7.0) and 0.5% NP-40) at 4°C and then centrifuged at 13,000 rpm for 10 min. The supernatants were collected and digested with RNase A and DNase I (Thermo Fisher) in the presence of DNase I buffer at 37°C for 2 h. DNase I was inactivated subsequently at 100°C for 5 min in the presence of 10 mM EDTA, and then proteinase K was used to digest the protein along with 1% SDS overnight at 55°C. Finally, the digested samples were extracted with phenol/chloroform. After that, DNA samples were precipitated with ethanol and then resolved in 30 μL Tris-EDTA (TE) buffer. The HBV core-associated DNA from the cells was then analyzed by Southern blotting or qPCR using the primers mentioned in S2 Table.

### Recovery of transfected DNA or rcccDNA

This assay was conducted according to a protocol described previously [57]. At the indicated time points, the transfected cells were lysed by Hirt lysis buffer (0.6% SDS/10 mM EDTA) and incubated at room temperature for 15 min. Then, the genomic DNA and cellular debris were precipitated by adding 5 M NaCl (incubated overnight) and centrifugation at 4°C, and the supernatants were transferred to fresh tubes. After phenol/chloroform extraction, ethanol precipitation and washing, the DNA pellets containing the recovered DNA were resuspended in Tris/EDTA buffer.

### Southern blotting

Southern blotting for HBV core-associated rcDNA and recovered DNA was conducted as described previously [52]. All of extracted DNA was loaded and separated in a 0.8% agarose gel. After standard denaturation and neutralization procedures, the DNA was transferred onto a positively charged nylon membrane (GE Healthcare, Waukesha, WI) and probed with a digoxigenin (DIG)-labeled HBV RNA probe. The probe preparation and subsequent DIG detection were conducted with the DIG Northern starter kit (Roche Diagnostics, Indianapolis, IN) according to the manufacturer’s instructions.

### Western blotting

Transfected cells were collected and lysed with NP40 lysis buffer (50 mM Tris-HCl, pH 7.0, 1% NP-40, and 150 mM NaCl). The mixture was separated on polyacrylamide gel with a gradient concentration, then transferred to a nitrocellulose membrane (0.45 μm, GE Healthcare). The membrane was blocked with 5% nonfat milk and incubated overnight at 4°C with primary antibody against NSUN2 (Proteintech, 20854-1-AP), HA (Sigma, H3663), beta-actin (Abclonal, AC026), GAPDH (Proteintech, 60004-1-Ig), and HBc (Gene Technology, GB058629). After washing 3 times, the membrane was incubated with goat anti-rabbit secondary antibody for 1 hour. Finally, the membrane was washed 3 times and then exposed with a ChemiDoc Imaging system (Bio-rad) after adding Chemiluminescent HRP Substrate (Millipore).

### RNA extraction and quantitative real-time PCR

The total RNA from cells was isolated using TRIzol reagent (Invitrogen). The cDNA was synthesized using a NovoScript 1st Strand cDNA Synthesis Kit (Novoprotein) after removing DNA. Quantitative real-time PCR (qPCR) was prepared with Hieff qPCR SYBR Green Master Mix (Yeasen) and performed with a CFX96 Real-Time PCR Detection System (Bio-rad). The mRNA of the housekeeping gene glyceraldehyde-3-phosphate dehydrogenase (*GAPDH*) functioned as an endogenous standard. All the primers used in qRT-PCR are listed in S2 Table.

For half-life determination, plasmids expressing HBV were transfected into Huh7 cells. Then, 36 hours later, actinomycin D (15 μg/mL, MedChemExpress) was added to inhibit RNA transcription. Then, cells were collected at 0, 3, 6, and 9 hours with TRIzol reagent for RNA extraction and qPCR. *GAPDH* was used as the internal reference.

### Northern blotting

All the procedures followed the instructions provided with the DIG Northern Starter Kit (Roche Diagnostics). In brief, 4 μg total RNA was separated using 1% denaturing agarose gel containing 2.2 M formaldehyde and transferred to an Amersham Hybond-N membrane (GE Healthcare) by capillary transfer. After UV cross-linking of the transferred RNA to the membrane and blocking with prehybridization solution at 60°C for 1 h, denatured DIG-labeled RNA probe was added for hybridizing overnight. Then, the membrane was blocked with blocking solution for 30 min and incubated with DIG antibody for 30 min. Finally, after adding CDP-Star (Roche), the membrane was exposed for 15 min with ChemiDoc MP (Bio-rad). The DIG-labeled plus strand-specific RNA probe corresponding to nucleotides 156–1061 of the HBV genome was used for HBV RNA detection [52]. We used 28S and 18S rRNA as loading controls.

### HBV viral infection

Viral infections of HBV were performed as previously described [58]. Huh7 was transfected with plasmids containing the HBV genome to produce the HBV genotype D virus. Virus titer was determined with qPCR after rcDNA extraction. HepG2-NTCP cells were plated on collagen-coated cell culture 96-well plates. At 4 hours after plating, the medium was changed to primary hepatocyte maintenance medium (PMM); that is, Williams E medium supplemented with 5 μg/mL transferrin, 10 ng/mL EGF, 3 μg/mL insulin, 2 mM L-glutamine, 18 μg/mL hydrocortisone, 40 ng/mL dexamethasone, 5 ng/mL sodium selenite, 2% DMSO, 100 U/mL penicillin, and 100 μg/mL streptomycin. After 24 hours, HBV virus was inoculated with cells in the presence of 5% PEG8000 and incubated for 16 hours, with a multiplicity of infection (MOI) of 400. Cells were then washed with medium 3 times and maintained in PMM medium. The medium was changed every 2–3 days. 3 and 9 days after infection, secreted HBsAg or HBeAg were determined with ELISA kits. HBV preC/pgRNA, total HBV RNA, HBV cccDNA, and HBV rcDNA levels were quantified with qPCR, with or without a prior reverse-transcription step. HBcAg was analyzed with Western blot. Primary human hepatocytes (PHHs) were cultured and infected according to the manufacturer’s protocol. 9 days after infection, culture supernatant and cells were harvested for analysis.

### HBV cccDNA exraction and analysis

Total DNA including HBV intracellular DNA and cccDNA from infected cells was extracted using a TIANamp Genomic DNA Kit (TIANGEN Biotech). DNA samples for cccDNA qPCR were treated with 500 U/mL T5 exonuclease (NEB) at 37°C for 30 min. A cccDNA-specific primer pair listed in S2 Table, cccDNA 92 fw/cccDNA 2251 rev, was used for qPCR [59].

### Immunofluorescence microscopy analysis

HBV transfected cells were fixed with 4% paraformaldehyde (PFA) and permeabilized with 0.2% TritonX-100. After blocking with 1% BSA, cells were stained with primary antibody against HBcAg (Dako) followed by Alexa-Fluor-labeled goat antirabbit IgG (Thermo Fisher). We added 1 μg/mL of Hoechst 33342 (Thermo Fisher) to stain the nucleus before analyzing. The cell images were captured with an Inverted Fluorescence Microscope (Leica).

### m^5^C-Methylated RNA immunoprecipitation qPCR (MeRIP-qPCR)

Total RNA from HBV transfected or infected cells was incubated with anti-m^5^C antibody (Abcam, ab10805) or anti-m^6^A antibody (Synaptic Systems, Germany) in 800 μL of IPP buffer (150 mM NaCl, 0.1% NP-40, 10 mM Tris–HCl, pH 7.4) for 2 h at 4°C. The mixture was then incubated with 30 μL proteinA/G beads (MedChemExpress) overnight at 4°C. The beads were then washed 5 times with IPP buffer, followed by RNA extraction and qPCR analysis.

### Mice experiment

C57BL/6JGpt-*Nsun2*^+/-^ mice were obtained from Gempharmatech Co.,Ltd (Nanjing, China) and housed and bred in specific pathogen-free conditions. The knockout targeting strategy is outlined in S6 Fig. For *in vivo* experiments, 4-week-old male mice were used and separated into four groups (six mice per group) based on genotyping results. Rapid genotyping was performed using the KAPA mouse genotyping kit (Kapa Biosystems), and the primers used for genotyping are shown in S2 Table. pHBV1.3 (15 μg) and its mutants were injected into the tail vein within 8 s in a volume of PBS equivalent to 10% of the mouse body weight. Animals were sacrificed 4 days after injection. Sera were taken for analysis of HBsAg, HBeAg and HBV DNA. For Northern blotting analysis, a piece of liver tissue was homogenized in 1 ml TRIzol reagent (Life Technologies), and total RNA was isolated following the manufacturer’s protocol. Total RNA (10 μg) of each mouse was subjected to Northern blotting analysis as described above. For Western blotting analysis, the liver tissues were homogenized in RIPA lysis buffer (Beyotime Biotechnology), then followed by standard protocols. Core antigen expression in mice livers was analyzed with immunohistochemical staining by Wuhan Pinuofei biological company following the usual methods with primary antibody against HBc (Dako, B0586).

### Data analysis

All experiments were repeated at least three times. The results are presented as means ± SD unless stated otherwise. The statistically significant differences were determined using Student’ s t-test for two groups and one- or two-way ANOVA analysis for more than two groups. Statistical analyses were achieved using Prism 8 software (GraphPad Software Inc.). *P* ≤ 0.05 was considered statistically significant.

## Supporting information

S1 Fig NSUN2 depletion in HepG2 negatively regulates HBV expression and replication. (A-D) pHBV1.3 and pSV-β-gal were transfected into HepG2 and HepG2-NSUN2-KO cells. At 48 hours later, supernatant was collected for ELISA. Cells were harvested for RNA or rcDNA extraction. β-galactosidase activity in cell lysates was measured to normalize efficiency of transfection for ELISA. (*A*) Western blot of NSUN2 protein for HepG2 and HepG2-NSUN2-KO cells. (*B*) ELISA results of HBV antigens after NSUN2 knockout in HepG2. (*C*) Northern blot (left) and gray degree (right) analysis of HBV RNA after NSUN2 knockout in HepG2. Ribosomal RNAs (28S and 18S) were used as the loading control. Relative levels of HBV RNA were quantified using Quantity One. (*D*) Southern blot (left) and gray degree analysis (right) of HBV core associated DNA after NSUN2 knockout in HepG2. RI, replication intermediates. (*E*) Western blot of NSUN2 protein for Huh7 and Huh7-NSUN2-KO cells with NSUN2 or NSUN2-I302A/321A rescue after 48 hours. Immunoblots shown are representative of three independent experiments. Graphs show the mean ± SD derived from three independent experiments and were analyzed by two-way ANOVA analysis (two targets) followed by multiple comparisons test. ns, not significant for P > 0.05, *P < 0.05, **P < 0.01, ***P< 0.001, ****P< 0.0001.
(TIF)

S2 Fig m^5^C methylated sites in HBV RNA detected via *in vitro* bisulfite sequencing. Multiple alignments of bisulfite-converted sequences of sites C2017, C2268, and C131 with Sanger sequencing. Ratios of unconverted reads to total reads are indicated. Fragment of HBe-2 without *in vitro* methylation was used as the negative control. The sequence at the bottom indicated with red arrow represented the original or reference sequence and was not included in the total read numbers.
(TIF)

S3 Fig Sequence alignments of HBV from high-throughput bisulfite sequencing or different HBV genotypes. (*A*) IGV alignments of the regions containing sites C131, C2017, and C2268 from two biological replicates of high-throughput bisulfite sequencing of HBV RNA. Reads were aligned to the bisulfite-converted HBV genome sequence (NC_003977.2). Red in bar chart indicates original Cs from reference genome were converted to Ts, and blue in bar chart indicates m^5^C methylated Cs were not converted. Conversion rates (methylation rate) are indicated with percentages. Y-axis indicates read depth. (*B*) Sequence alignment of the regions containing sites C131, C2017, and C2268 from different HBV genotypes, with GenBank accession numbers. Red boxes indicate site positions.
(TIF)

S4 Fig m^5^C modified sites contributes to HBV replication in cccDNA-like system. (*A*) Schematic diagram of the prcccDNA/pCMV-Cre system, which utilizes the Cre recombinase to generate a surrogate for cccDNA known as rcccDNA. C2017A and C2268T mutations were introduced into the corresponding regions, respectively. (B and C) prcccDNA and pCMV-Cre were transfected into HepG2 cells. At 48 hours later, supernatant was collected for ELISA. Cells were harvested for RNA, rcccDNA, and rcDNA extraction. (*B*) Southern blot result of rcccDNA (top), Northern blot result (medium) of HBV RNA, and Southern blot (bottom) result of HBV rcDNA. RI, replication intermediates. (*C*) ELISA results of HBV antigens after site mutations. Graphs show the mean ± SD derived from three independent experiments and were analyzed by two-way ANOVA analysis (two targets) followed by multiple comparisons test. ns, not significant for P > 0.05, ****P< 0.0001.
(TIF)

S5 Fig m^5^C methylation in HBV RNA contributes to HBV RNA stability. (*A*) Half-life of HBV preC/pgRNA after TET2 depletion in Huh7. The expression of TET2 in Huh7 was depleted with siRNA, followed by transfection with pHBV1.3. At 36 hours later, actinomycin D (ActD, 15ug/ml) was added, and then cells were harvested after 0, 4, 8, 12, and 16 hours for qPCR with GAPDH as the control. The TET2 mRNA level was also determined with qPCR for assessing knockdown efficiency. (*B*) MeRIP-qPCR of m^5^C methylated HBV transcripts in Huh7 after TET2 depletion. FAM129b and HPRT1 serve as positive and negative controls, respectively. (*C*) Northern blot (left) and gray degree analysis (right) of HBV genomic and subgenomic RNA after NSUN2 knockout in Huh7. Graphs show the mean ± SD derived from three independent experiments and were analyzed by unpaired Student’s t test. ns, not significant for P > 0.05, **P < 0.01, ***P< 0.001, ****P< 0.0001.
(TIF)

S6 Fig The knockout strategy of *Nsun2* in C57BL/6JGpt mice and genotyping strategy. (*A*) *Nsun2*-202 transcript was used for sgRNA designing, and exon 4 - exon 9 was the targeting region with 8954 bp fragment deleted. (*B*) Two sets of primers were used for genotyping. (*C*) The size of targeted band is shown. For primer set F1/R1, if the WT band is too large, it may not be possible to obtain a WT band. According to all the genotyping results, no homozygote mice was detected. (*D*) Sequence alignment of *Nsun2*-202 transcript and PCR product using primer F1/R1 from Nsun2 heterozygote mice.
(TIF)

S1 Table The methylation levels of another two m^5^C methylated sites. (DOCX)

S2 Table CRISPR/CAS9 gRNA sequence and Primers for mRNA detection, HBV DNA detection and site mutation. (DOCX)

S3 Table Primers for *in vitro* methylation and bisulfite sequencing. (DOCX)

S4 Table Methylated sites on HBV RNA detected by *in vitro* bisulfite sequencing. (DOCX)
