# Supplementary figures and images for: NSUN2-mediated m5C modification of HBV RNA positively regulates HBV replication
Source: PLoS Pathog. 2023 Dec 4;19(12):e1011808. doi: 10.1371/journal.ppat.1011808 (PMC10721180; doi:10.1371/journal.ppat.1011808)

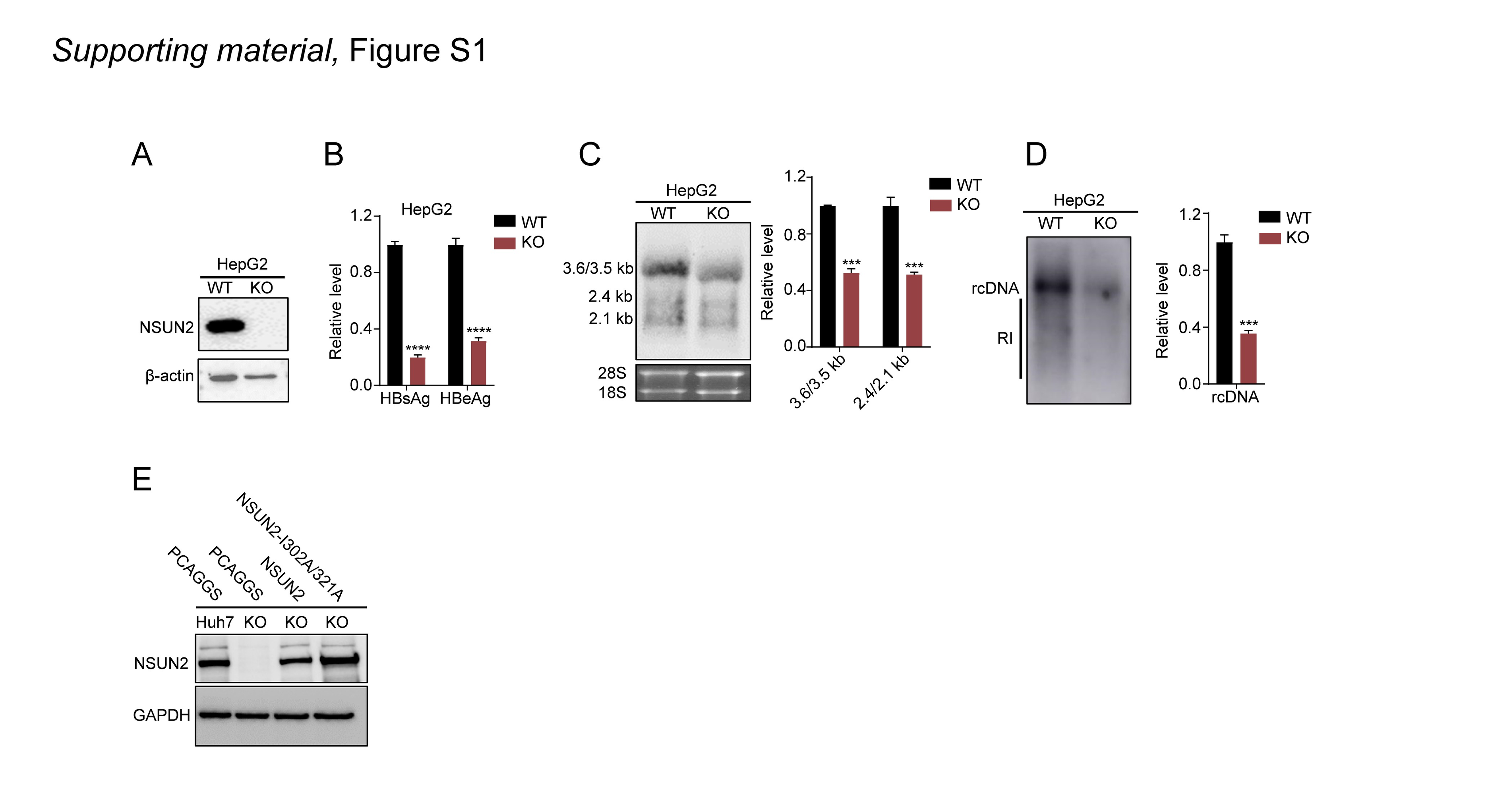

Supplement: S1 Fig — (A-D) pHBV1.3 and pSV-β-gal were transfected into HepG2 and HepG2-NSUN2-KO cells. At 48 hours later, supernatant was collected for ELISA. Cells were harvested for RNA or rcDNA extraction. β-galactosidase activity in cell lysates was measured to normalize efficiency of transfection for ELISA. (A) Western blot of NSUN2 protein for HepG2 and HepG2-NSUN2-KO cells. (B) ELISA results of HBV antigens after NSUN2 knockout in HepG2. (C) Northern blot (left) and gray degree (right) analysis of HBV RNA after NSUN2 knockout in HepG2. Ribosomal RNAs (28S and 18S) were used as the loading control. Relative levels of HBV RNA were quantified using Quantity One. (D) Southern blot (left) and gray degree analysis (right) of HBV core associated DNA after NSUN2 knockout in HepG2. RI, replication intermediates. (E) Western blot of NSUN2 protein for Huh7 and Huh7-NSUN2-KO cells with NSUN2 or NSUN2-I302A/321A rescue after 48 hours. Immunoblots shown are representative of three independent experiments. Graphs show the mean ± SD derived from three independent experiments and were analyzed by two-way ANOVA analysis (two targets) followed by multiple comparisons test. ns, not significant for P > 0.05, *P < 0.05, **P < 0.01, ***P< 0.001, ****P< 0.0001. (TIF) [file ppat.1011808.s001.tif]

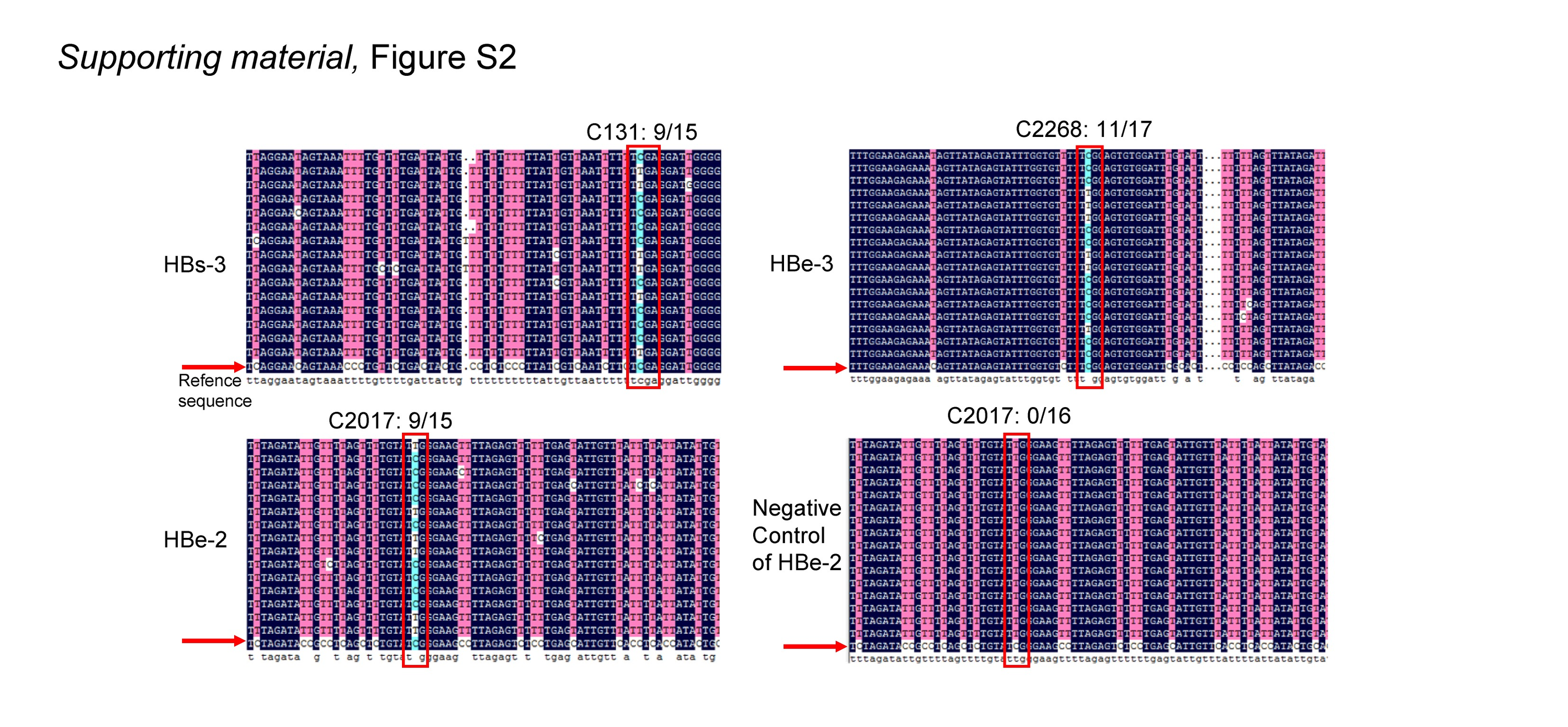

Supplement: S2 Fig — Multiple alignments of bisulfite-converted sequences of sites C2017, C2268, and C131 with Sanger sequencing. Ratios of unconverted reads to total reads are indicated. Fragment of HBe-2 without in vitro methylation was used as the negative control. The sequence at the bottom indicated with red arrow represented the original or reference sequence and was not included in the total read numbers. (TIF) [file ppat.1011808.s002.tif]

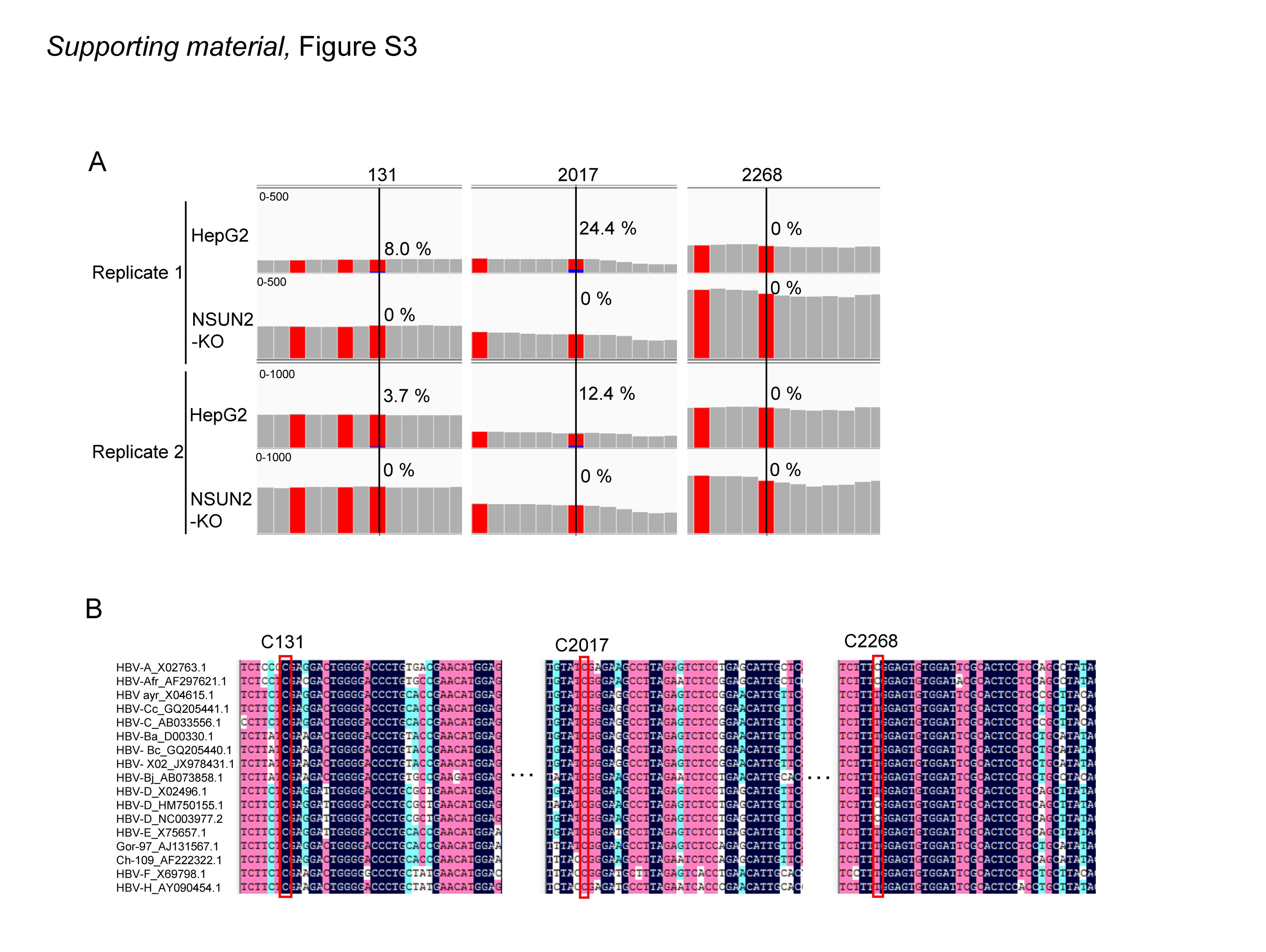

Supplement: S3 Fig — (A) IGV alignments of the regions containing sites C131, C2017, and C2268 from two biological replicates of high-throughput bisulfite sequencing of HBV RNA. Reads were aligned to the bisulfite-converted HBV genome sequence (NC_003977.2). Red in bar chart indicates original Cs from reference genome were converted to Ts, and blue in bar chart indicates m5C methylated Cs were not converted. Conversion rates (methylation rate) are indicated with percentages. Y-axis indicates read depth. (B) Sequence alignment of the regions containing sites C131, C2017, and C2268 from different HBV genotypes, with GenBank accession numbers. Red boxes indicate site positions. (TIF) [file ppat.1011808.s003.tif]

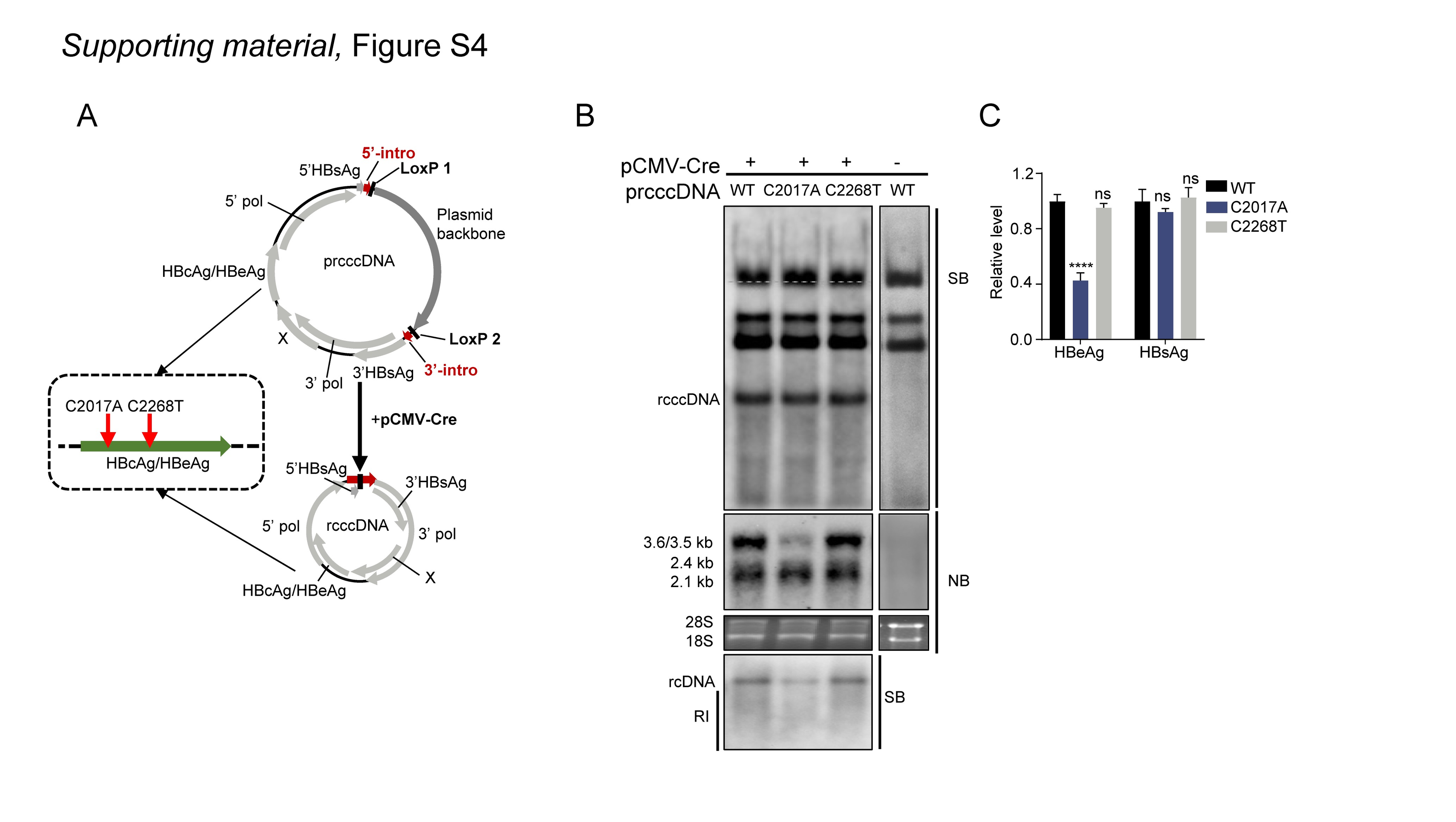

Supplement: S4 Fig — (A) Schematic diagram of the prcccDNA/pCMV-Cre system, which utilizes the Cre recombinase to generate a surrogate for cccDNA known as rcccDNA. C2017A and C2268T mutations were introduced into the corresponding regions, respectively. (B and C) prcccDNA and pCMV-Cre were transfected into HepG2 cells. At 48 hours later, supernatant was collected for ELISA. Cells were harvested for RNA, rcccDNA, and rcDNA extraction. (B) Southern blot result of rcccDNA (top), Northern blot result (medium) of HBV RNA, and Southern blot (bottom) result of HBV rcDNA. RI, replication intermediates. (C) ELISA results of HBV antigens after site mutations. Graphs show the mean ± SD derived from three independent experiments and were analyzed by two-way ANOVA analysis (two targets) followed by multiple comparisons test. ns, not significant for P > 0.05, ****P< 0.0001. (TIF) [file ppat.1011808.s004.tif]

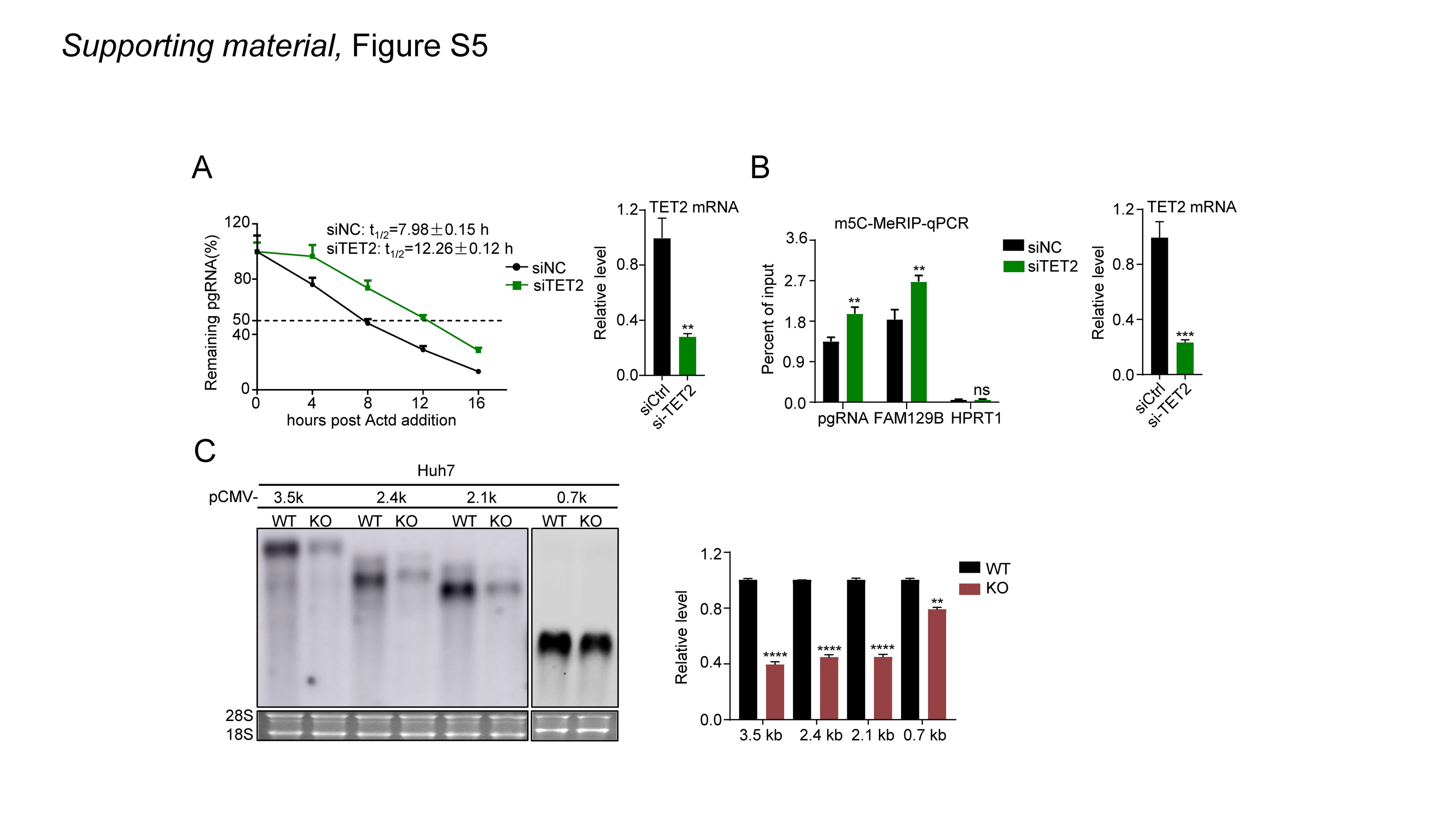

Supplement: S5 Fig — (A) Half-life of HBV preC/pgRNA after TET2 depletion in Huh7. The expression of TET2 in Huh7 was depleted with siRNA, followed by transfection with pHBV1.3. At 36 hours later, actinomycin D (ActD, 15ug/ml) was added, and then cells were harvested after 0, 4, 8, 12, and 16 hours for qPCR with GAPDH as the control. The TET2 mRNA level was also determined with qPCR for assessing knockdown efficiency. (B) MeRIP-qPCR of m5C methylated HBV transcripts in Huh7 after TET2 depletion. FAM129b and HPRT1 serve as positive and negative controls, respectively. (C) Northern blot (left) and gray degree analysis (right) of HBV genomic and subgenomic RNA after NSUN2 knockout in Huh7. Graphs show the mean ± SD derived from three independent experiments and were analyzed by unpaired Student’s t test. ns, not significant for P > 0.05, **P < 0.01, ***P< 0.001, ****P< 0.0001. (TIF) [file ppat.1011808.s005.tif]

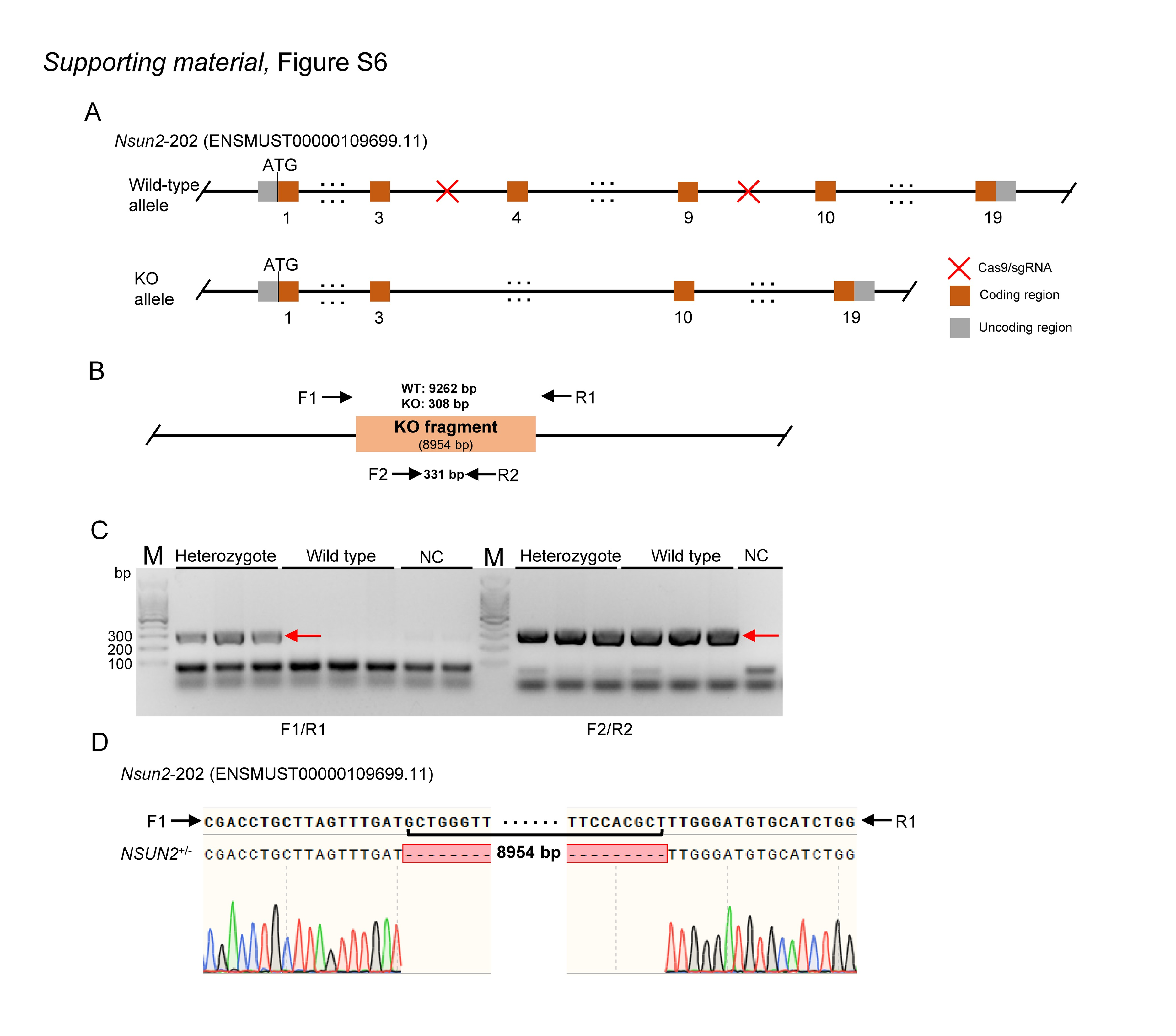

Supplement: S6 Fig — (A) Nsun2-202 transcript was used for sgRNA designing, and exon 4 - exon 9 was the targeting region with 8954 bp fragment deleted. (B) Two sets of primers were used for genotyping. (C) The size of targeted band is shown. For primer set F1/R1, if the WT band is too large, it may not be possible to obtain a WT band. According to all the genotyping results, no homozygote mice was detected. (D) Sequence alignment of Nsun2-202 transcript and PCR product using primer F1/R1 from Nsun2 heterozygote mice. (TIF) [file ppat.1011808.s006.tif]
