# Supplementary material for: NSUN2-mediated m5C modification of HBV RNA positively regulates HBV replication
Source: PLoS Pathog. 2023 Dec 4;19(12):e1011808. doi: 10.1371/journal.ppat.1011808 (PMC10721180; doi:10.1371/journal.ppat.1011808)
Supplement: S1 Table — (DOCX) [file ppat.1011808.s007.docx]

**S1_Table. The methylation levels of another two m^5^C methylated sites.**

|  | WT-1 | WT-2 | KO-1 | KO-2 |
| --- | --- | --- | --- | --- |
| C173 | 3.4% | 4.0% | 0% | 0% |
| C224 | 4.8% | 3.9% | 0% | 0% |

WT: HepG2-WT, KO: HepG2-NSUN2-KO.
