# Supplementary material for: NSUN2-mediated m5C modification of HBV RNA positively regulates HBV replication
Source: PLoS Pathog. 2023 Dec 4;19(12):e1011808. doi: 10.1371/journal.ppat.1011808 (PMC10721180; doi:10.1371/journal.ppat.1011808)
Supplement: S2 Table — (DOCX) [file ppat.1011808.s008.docx]

**S2_Table. Crispr/Cas9 gRNA sequence and Primers for mRNA detection, HBV DNA detection and site mutation.**

|  | **Crispr/Cas9 gRNA** |
| --- | --- |
| NSUN2-gRNA | GACGCGGAGGATGGCGCCGA |
|  | shRNA for knockdown |
| NSUN2-shRNA | CGGCCTCATCATAAGATCTTAGATA |
| Control-shRNA | AAGTGTAGTAGATCACCAGGC |
|  | **Primers for mRNA detection** |
| HBV-pgRNA-F | CTCCTCCAGCTTATAGACC |
| HBV-pgRNA-R | GTGAGTGGGCCTACAAA |
| total HBV RNA-F | CACCAGCACCATGCAAC |
| total HBV RNA-R | AAGCCACCCAAGGCACAG |
| GAPDH-F | TGCACCACCAACTGCTTAGC |
| GAPDH-R | GGCATGGACTGTGGTCATGAG |
| qNSUN2-F | GCTACCCCGAGATCGTCAAG |
| qNSUN2-R | CTTTCCTGCCGTCCACATCT |
| qDNMT2-F | CAGTAGTTGAAGAAAGTCAGCCA |
| qDNMT2-R | AGCCTGTAACACGGAGCCTG |
| qMETLL3-F | CTATCTCCTGGCACTCGCAAGA |
| qMETLL3-R | GCTTGAACCGTGCAACCACATC |
| qMETLL14-F | CTGAAAGTGCCGACAGCATTGG |
| qMETLL14-R | CTCTCCTTCATCCAGATACTTACG |
| qYBX1-F | GCAGGAGAACAAGGTAGACCAG |
| qYBX1-R | CTTCATTGCCGTCCTCTCTAGG |
| qALYREF-F | GGAGTCTCAGACGCCGATATTC |
| qALYREF-R | GCATCTGCCTTCCGCTCAAAGT |
| qTET2-F | GCTTACCGAGACGCTGAGGAAA |
| qTET2-R | AGAGAAGGAGGCACCACAGGTT |
| hFAM129B-F | CTTCAAGGAGGTCACGGACATG |
| hFAM129B-R | CGACTCCATCTTCTCATAGCAGC |
| hHPRT1-F | TGACACTGGCAAAACAATGCA |
| hHPRT1-R | GGTCCTTTTCACCAGCAAGCT |
| hCREBBP-F | ACAAGCGAAACCAACAAACC |
| hCREBBP-R | CCTGTGCCAACAGAACCAA |
|  | **Primers for HBV DNA detection** |
| HBV DNA-F | CTCGTGGTGGACTTCTCTC |
| HBV DNA-R | CTGCAGGATGAAGAGGAA |
| cccDNA 92 fw | GCCTATTGATTGGAAAGTATGT |
| cccDNA 2251 rev | AGCTGAGGCGGTATCTA |
|  | **Primers for site mutation** |
| C131T-F | CAATCTTCTTGAGGATTGGGGACCCTG |
| C131T-R | CCAATCCTCAAGAAGATTGACGATAAG |
| C2017A-F | CAGCTCTGTATAGGGAAGCCTTAGAGTCTC |
| C2017A-R | GGCTTCCCTATACAGAGCTGAGGCGGTATC |
| C2268T-F | GGTGTCTTTTGGAGTGTGGATTCGCACTC |
| C2268T-R | CACACTCCAAAAGACACCAAATACTC |
| C173T-F | GAACATCACATTAGGATTCCTAGGAC |
| C173T-R | GGAATCCTAATGTGATGTTCTCCATG |
| C224T-F | CTTGTTGATAAGAATCCTCACAATAC |
| C224T-R | GGATTCTTATCAACAAGAAAAACC |
| A1907C-F | TGGGGCATGGCCATCGACCCTTATAAAG |
| A1907C-R | GGGTCGATGGCCATGCCCCAAAGCCAC |
|  | **Primers for genotyping of C57BL/6JGpt-*Nsun*2^+/-^ mice** |
| F1 | CAGTCAGCCTGATTTCATTTTCCC |
| R1 | TTTACAGCAGTGGGACGGGTAC |
| F2 | AACGGGCCTGCACATAGTACAGAC |
| R2 | AAACCGTTCCTGCCAGAACTGG |
