# Supplementary material for: NSUN2-mediated m5C modification of HBV RNA positively regulates HBV replication
Source: PLoS Pathog. 2023 Dec 4;19(12):e1011808. doi: 10.1371/journal.ppat.1011808 (PMC10721180; doi:10.1371/journal.ppat.1011808)
Supplement: S3 Table — (DOCX) [file ppat.1011808.s009.docx]

**S3_Table. Primers for *in vitro* methylation and bisulfite sequencing**

|  | **Primers for RNA *in vitro* methylation** |
| --- | --- |
| pgRNA-F | TAATACGACTCACTATAGGGTCGGGAAGCCTTAGAG |
| pgRNA-R | TGAAGGAAAGAAGTCAGAA |
| F1-F | TAATACGACTCACTATAGGGGAACATCACATCAGGATTCCTAG |
| F1-R | TGCTTAGATTGAATACATGCATACA |
| F2-F | TAATACGACTCACTATAGGGTCAATCTAAGCAGGCTTTCACTT |
| F2-R | AGTGTTGACATAACTGACTACTAGG |
| F3-F | TAATACGACTCACTATAGGGTATGTCAACACTAATATGGGC |
| F3-R | TCCTAGGAATCCTGATGTGAT |
|  | **Primers for *in vitro* bisulfite sequencing** |
| m5C_adaptor-F | AGGTTTGGTTGAAGTTGA |
| m5C_adaptor-R | ATACCTCCATAACCATTT |
| E1-F | TAATACGACTCACTATAGGGAGGTCTGGCTGAAGTTGAATGCAACTTTTTCACCTCTG |
| E1-R | ATACCTCCGTGACCATTTCTAGAAGATCTCGTACTGAAGG |
| E2-F | TAATACGACTCACTATAGGGAGGTCTGGCTGAAGTTGACTGACTTCTTTCCTTCAGTAC |
| E2-R | ATACCTCCGTGACCATTTGACGCTGGATCTTCCAAATTAAC |
| E3-F | TAATACGACTCACTATAGGGAGGTCTGGCTGAAGTTGATTAATTTGGAAGATCCAGCGT |
| E3-R | ATACCTCCGTGACCATTTGGTCTATAAGCTGGAGGAGTGCG |
| E4-F | TAATACGACTCACTATAGGGAGGTCTGGCTGAAGTTGAGTGGATTCGCACTCCTCC |
| E4-R | ATACCTCCGTGACCATTTCTAACATTGAGATTCCCGAGATTG |
| PreS1-F | TAATACGACTCACTATAGGGAGGTCTGGCTGAAGTTGAAAACCTTATTATCCAGAAC |
| PreS1-R | ATACCTCCGTGACCATTTTTCTGCCCCATGCTGTAG |
| S1-F | TAATACGACTCACTATAGGGAGGTCTGGCTGAAGTTGAATGGGGCAGAATCTTTCCACC |
| S1-R | ATACCTCCGTGACCATTTTGAGGGCTCCACCCCAAAAGGC |
| S2-F | TAATACGACTCACTATAGGGAGGTCTGGCTGAAGTTGAGCCTTTTGGGGTGGAGCCCTC |
| S2-R | ATACCTCCGTGACCATTTCCACCAGCAGGGAAATACAGGCC |
| S3-F | TAATACGACTCACTATAGGGAGGTCTGGCTGAAGTTGAGCCTGTATTTCCCTGCTGGTG |
| S3-R | ATACCTCCGTGACCATTTCACCACGAGTCTAGACTCTGCG |
| S4-F | TAATACGACTCACTATAGGGAGGTCTGGCTGAAGTTGACGCAGAGTCTAGACTCGTGG |
| S4-R | ATACCTCCGTGACCATTTTGATAGTCCAGAAGAACCAAC |
| S5-F | TAATACGACTCACTATAGGGAGGTCTGGCTGAAGTTGATGTTGGTTCTTCTGGACTATC |
| S5-R | ATACCTCCGTGACCATTTGCCCACTCCCATAGGAATTTTC |
| S6-F | TAATACGACTCACTATAGGGAGGTCTGGCTGAAGTTGAGAAAATTCCTATGGGAGTGGG |
| S6-R | ATACCTCCGTGACCATTTTTAAATGTATACCCAAAGAC |
| X1-F | TAATACGACTCACTATAGGGAGGTCTGGCTGAAGTTGAGGAACCTTTTCGGCTCCTCTG |
| X1-R | ATACCTCCGTGACCATTTTTCAGCGCCGACGGGACGTAAAC |
| X2-F | TAATACGACTCACTATAGGGAGGTCTGGCTGAAGTTGATTTACGTCCCGTCGGCGCTG |
| X2-R | ATACCTCCGTGACCATTTCAATATTTGGTGGGCGTTCACG |
| X3-F | TAATACGACTCACTATAGGGAGGTCTGGCTGAAGTTGAGTGAACGCCCACCAAATATTG |
| X3-R | ATACCTCCGTGACCATTTAAAAGTTGCATGGTGCTGGTG |
| P-1-F | TAATACGACTCACTATAGGGAGGTCTGGCTGAAGTTGATAGTATTCCTTGGACTCATAAG |
| P-1-R | ATACCTCCGTGACCATTTATAATAAGGTTTAATACCCTTATC |
| P-2-F | TAATACGACTCACTATAGGGAGGTCTGGCTGAAGTTGATGGGTATACATTTAAACCCTAAC |
| P-2-R | ATACCTCCGTGACCATTTGCAGGATAACCACATTGTGTAAAAGG |
| P-3-F | TAATACGACTCACTATAGGGAGGTCTGGCTGAAGTTGATTACACAATGTGGTTATCCTGC |
| P-3-R | ATACCTCCGTGACCATTTGAGCCGAAAAGGTTCCACGCATG |
