# Supplementary material for: NSUN2-mediated m5C modification of HBV RNA positively regulates HBV replication
Source: PLoS Pathog. 2023 Dec 4;19(12):e1011808. doi: 10.1371/journal.ppat.1011808 (PMC10721180; doi:10.1371/journal.ppat.1011808)
Supplement: S4 Table — (DOCX) [file ppat.1011808.s010.docx]

**S4_Table. Methylated sites on HBV RNA detected by *in vitro* bisulfite sequencing.**

| HBV  Fragments | NC_003977.2  Site location* | Methylation Rate | Reads ratio |
| --- | --- | --- | --- |
| HBs3 | 71 | 33.30% | 5/15 |
| HBs3 | 131 | 60.00% | 9/15 |
| HBs3 | 156 | 20.00% | 3/15 |
| HBs4 | 314 | 22.20% | 2/9 |
| HBs4 | 356 | 22.20% | 2/9 |
| HBs4 | 375 | 22.20% | 2/9 |
| HBs5 | 445 | 18.20% | 2/11 |
| HBs5 | 630 | 27.30% | 3/11 |
| HBs6 | 661 | 14.30% | 2/14 |
| HBs6 | 799 | 25.00% | 2/8 |
| HBp2 | 845 | 14.30% | 2/14 |
| HBp2 | 1033 | 21.40% | 3/14 |
| HBp3 | 1102 | 13.30% | 2/15 |
| HBp3 | 1112 | 40.00% | 6/15 |
| HBp3 | 1151 | 13.30% | 2/15 |
| HBp3 | 1195 | 13.30% | 2/15 |
| HBp3 | 1252 | 20.00% | 3/15 |
| HBx-1 | 1328 | 35.29% | 6/17 |
| HBx-1 | 1354 | 17.65% | 3/17 |
| HBx-2 | 1500 | 28.57% | 4/14 |
| HBx-3 | 1627 | 25.00% | 4/16 |
| HBx-3 | 1645 | 18.75% | 3/16 |
| HBe1 | 1914 | 18.80% | 3/16 |
| HBe1 | 1937 | 18.80% | 3/16 |
| HBe2 | 2017 | 60.00% | 9/15 |
| HBe3 | 2268 | 64.71% | 11/17 |
| HBs1 | 2973 | 23.10% | 3/13 |
| HBs2 | 3035 | 14.30% | 2/14 |

* **Only Cs with unconverted reads were calculated here.**
